# Nab3 Facilitates the Function of the TRAMP Complex in RNA Processing via Recruitment of Rrp6 Independent of Nrd1

**DOI:** 10.1371/journal.pgen.1005044

**Published:** 2015-03-16

**Authors:** Milo B. Fasken, R. Nicholas Laribee, Anita H. Corbett

**Affiliations:** 1 Department of Biochemistry, Emory University School of Medicine, Atlanta, Georgia, United States of America; 2 Department of Pathology and Laboratory Medicine and Center for Adult Cancer Research,University of Tennessee Health Science Center, Memphis, Tennessee, United States of America; University of Rochester Medical Center, UNITED STATES

## Abstract

Non-coding RNAs (ncRNAs) play critical roles in gene regulation. In eukaryotic cells, ncRNAs are processed and/or degraded by the nuclear exosome, a ribonuclease complex containing catalytic subunits Dis3 and Rrp6. The TRAMP (Trf4/5-Air1/2-Mtr4 polyadenylation) complex is a critical exosome cofactor in budding yeast that stimulates the exosome to process/degrade ncRNAs and human TRAMP components have recently been identified. Importantly, mutations in exosome and exosome cofactor genes cause neurodegenerative disease. How the TRAMP complex interacts with other exosome cofactors to orchestrate regulation of the exosome is an open question. To identify novel interactions of the TRAMP exosome cofactor, we performed a high copy suppressor screen of a thermosensitive *air1/2* TRAMP mutant. Here, we report that the Nab3 RNA-binding protein of the Nrd1-Nab3-Sen1 (NNS) complex is a potent suppressor of TRAMP mutants. Unlike Nab3, Nrd1 and Sen1 do not suppress TRAMP mutants and Nrd1 binding is not required for Nab3-mediated suppression of TRAMP suggesting an independent role for Nab3. Critically, Nab3 decreases ncRNA levels in TRAMP mutants, Nab3-mediated suppression of *air1/2* cells requires the nuclear exosome component, Rrp6, and Nab3 directly binds Rrp6. We extend this analysis to identify a human RNA binding protein, RALY, which shares identity with Nab3 and can suppress TRAMP mutants. These results suggest that Nab3 facilitates TRAMP function by recruiting Rrp6 to ncRNAs for processing/degradation independent of Nrd1. The data raise the intriguing possibility that Nab3 and Nrd1 can function independently to recruit Rrp6 to ncRNA targets, providing combinatorial flexibility in RNA processing.

## Author Summary

Eukaryotic genomes from yeast to man express numerous non-coding RNAs (ncRNAs) that regulate the expression of messenger RNAs (mRNAs) encoding the proteins vital for cell and body function. As faulty ncRNAs impair mRNA expression and contribute to cancers and neurodegenerative disease, it is imperative to understand how ncRNAs are processed and/or degraded. In budding yeast, a conserved RNA shredding machine known as the exosome nibbles at or destroys ncRNAs. The exosome is assisted by a conserved TRAMP exosome cofactor that recruits the exosome to ncRNAs for processing/ degradation. To better understand TRAMP function, we performed a genetic screen to identify genes that improve the growth of TRAMP mutant yeast cells that grow poorly at high temperature. We find that overexpression of the Nab3 RNA binding protein, which belongs to another exosome cofactor, the Nrd1-Nab3-Sen1 (NNS) complex, improves the growth of TRAMP mutant cells. Importantly, Nab3 requires the exosome to improve the growth and ncRNA processing of TRAMP mutant cells. We therefore suggest that Nab3 facilitates TRAMP function by recruiting the exosome to ncRNAs for processing/degradation. We also show that the human RNA binding protein, RALY, like Nab3, can improve the growth of TRAMP mutant cells.

## Introduction

Non-coding RNAs (ncRNAs) are fast emerging as key regulators of gene expression in eukaryotic cells [1,2]. Dysregulation of ncRNAs is linked to cancer and neurodegeneration [3,4]. Understanding how ncRNAs are produced, processed, and destroyed is therefore critical to elucidating gene regulation and has become an area of major research focus in recent years. Transcriptional termination and 3’-end processing of ncRNAs are functionally linked but how they are coupled is poorly characterized [5]. In *Saccharomyces cerevisiae*, unlike most mRNAs, ncRNAs are not terminated and 3’-end processed by the conventional cleavage and polyadenylation machinery, but by a trio of evolutionarily conserved complexes: the RNA exosome, the TRAMP complex, and the NNS complex [5]. Critically, the interactions and mechanisms employed by these complexes to terminate and process/degrade ncRNAs are not well understood.

In budding yeast, ncRNAs are processed and/or degraded by the evolutionarily conserved ribonuclease complex, the RNA exosome [6,7]. The nuclear and cytoplasmic core exosome, which is composed of ten essential subunits, including the key catalytic 3’-5’-riboexonuclease Dis3/Rrp44, forms a ring-shaped structure through which RNA is threaded for processing or degradation [6,7]. The nuclear exosome contains an additional key nuclear catalytic subunit, the 3’-5’-riboexonuclease, Rrp6 [6,7]. The nuclear exosome processes and/or degrades several classes of ncRNAs, including rRNAs, snRNAs, snoRNAs, and tRNAs, as well as some mRNAs [6,7]. In addition, the nuclear exosome rapidly degrades a novel class of ncRNAs, termed cryptic unstable transcripts (CUTs) [8].

The exosome is recruited to RNAs by exosome cofactors that recognize the RNA and regulate exosome function [9,10]. Certain exosome cofactors also facilitate transcriptional termination of ncRNAs [5,10]. Exosome cofactors are usually single RNA binding proteins or protein complexes containing RNA binding proteins that recognize RNAs in a sequence or structure-specific manner and interact with core exosome and/or Rrp6 [5,10]. Three examples of exosome cofactors in *Saccharomyces cerevisiae* are the Rrp47 protein, the TRAMP complex, and the Nrd1-Nab3-Sen1 (NNS) complex [9,10].

The TRAMP (Trf4/5-Air1/2-Mtr4 Polyadenylation) complex is a key nuclear exosome cofactor that oligoadenylates the 3’-ends of ncRNAs and recruits the exosome to process/degrade these RNAs [11–13]. TRAMP is composed of a non-canonical poly(A) polymerase, Trf4 or Trf5, a zinc-knuckle RNA-binding protein, Air1 or Air2, and an essential RNA helicase, Mtr4 [11–13]. TRAMP targets include ncRNAs, such as snoRNAs and CUTs, and some mRNAs [11–13]. Air1 and Air2, which are critical for TRAMP assembly and function, contain five CCHC zinc knuckles that bind to RNA and Trf4 [11–16]. While *t*Δ*rག*, *trf5*Δ, *air1*Δ, and *air2*Δ single mutants are viable, the *trf4*Δ*tr*Δdouble mutant is inviable and the *trf4*Δsingle mutant and *air1*Δ*air2*Δ double mutant are growth impaired, indicating that Trf and Air activity is critical for cell function [13,16,17]. Importantly, human orthologues of Trf4/5, PAPD5/hTRF4–2, Air1/2, ZCCHC7, and Mtr4, hMTR4, have been identified, suggesting a TRAMP-like complex also functions in human cells [16,18].

The NNS (Nrd1-Nab3-Sen1) complex is a second nuclear exosome cofactor, composed of two sequence-specific RNA binding proteins, Nrd1 and Nab3, and an RNA/DNA ATP-dependent helicase, Sen1, that interacts with RNA polymerase II (RNA Pol II) and the exosome to stimulate termination and processing/degradation of ncRNAs [19–22]. The NNS subunits bind to different classes of ncRNAs, including snoRNAs, CUTs, tRNAs, and mRNAs, via NNS-dependent terminators [23,24]. All NNS components are essential, but *nrd1*, *nab3*, and *sen1* mutants show transcriptional readthrough of NNS terminators in ncRNAs and elevated levels of CUTs [20,21,25–28]. Nrd1 and Nab3 are single RNA recognition motif (RRM)-containing proteins that recognize the RNA sequences GUAG/A and UCUU, respectively, in the NNS terminators [21]. Nrd1 and Nab3 also directly interact with one another via Nab3/Nrd1-binding domains [29,30]. In addition, Nrd1 coprecipitates with the exosome and TRAMP and Nab3 interacts with Sen1 [19,31]. Specifically, Nrd1 directly interacts Rrp6 and Trf4 [32].

In current models for NNS function, Nrd1 binds to the carboxy-terminal domain of RNA Pol II during early transcription of a ncRNA and the Nrd1-Nab3 heterodimer recognizes the ncRNA terminator, recruits the Sen1 helicase to dissociate the elongation complex and terminate transcription in a mechanism involving Sen1 RNA binding and ATP hydrolysis, and recruits the exosome to process/degrade the ncRNA [5,19,29,30,33,34]. Notably, the human Sen1 orthologue, Senataxin, associated with neurodegenerative disorders, promotes termination in human cells, suggesting human counterparts of Nrd1 and Nab3 could exist [35–37].

To better understand the termination/processing of ncRNAs by exosome cofactors and the exosome, cryptic unstable transcripts (CUTs) have become important model ncRNA substrates. CUTs were first identified in budding yeast because they show elevated levels upon deletion of the gene encoding the nuclear exosome subunit, Rrp6 [13]. CUTs are short RNA Pol II-derived transcripts that can regulate gene expression by transcriptional interference or chromatin modification [8]. Two of the best-characterized CUTs are the *NEL025c* CUT and the *IMD2* CUT which contain Nrd1/Nab3 binding sites in their NNS terminators and are terminated/degraded by the NNS complex, TRAMP and the exosome [8,25–28,38]. Importantly, *NEL025c* and *IMD2* CUT levels and terminator readthrough are increased in *nrd1*, *nab3*, *trf4*Δ, and *rrp6*Δmutants [8,25–28,38]. In mammalian cells, novel ncRNAs termed promoter upstream transcripts (PROMPTs) that share similarity with CUTs are also degraded by the exosome [39].

The interactions between exosome cofactors and the exosome that are required to orchestrate proper termination/processing of ncRNAs are not well characterized. Determining the specific and shared functions of exosome cofactors and whether components of exosome cofactor complexes function only within the complex or can function separately are key areas of current investigation. To provide insight into TRAMP/exosome function, we performed a high copy suppressor screen with a thermosensitive *air1/2* TRAMP mutant [16]. Here, we report that Nab3 of the NNS complex is a potent suppressor of *air1/2* and *trf4*ΔTRAMP mutants. Our results suggest that Nab3 facilitates TRAMP function by recruiting Rrp6 to ncRNAs for processing/degradation independent of Nrd1. We also find that the human RNA binding protein, RALY, which shares identity with Nab3, can suppress TRAMP mutants. Combined, these data raise the intriguing possibility that Nab3 and Nrd1 RNA binding proteins may function independently to recruit the exosome to specific ncRNA targets, providing combinatorial flexibility in RNA processing dependent on the number of Nab3/Nrd1 binding sites.

## Results

### *NAB3* suppresses the thermosensitive growth of *air1/2* cells

**Fig 1:**
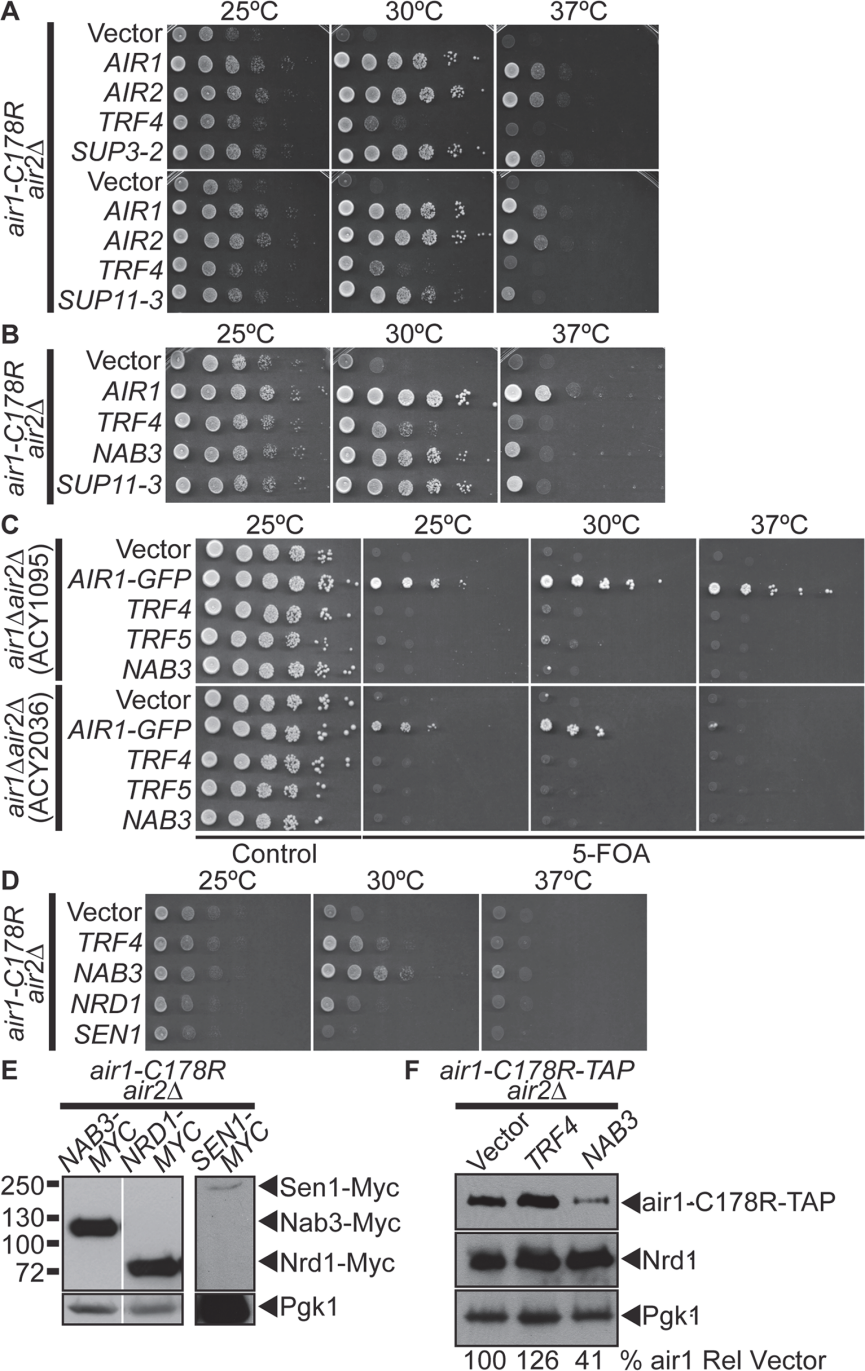
*NAB3* suppresses, but *NRD1* and *SEN1* do not suppress, *air1/2* thermosensitive growth. (**A**) A high copy suppressor screen identifies suppressor plasmid, *SUP11–3*, containing *NAB3*, as suppressor of *air1-C178R air2*Δthermosensitive growth at 30°C. The screen identified forty-four *AIR1/2* suppressor plasmids, including *SUP3–2* (*AIR1*). *NAB3* suppressor plasmid, *SUP11–3* (*NAB3*), suppresses *air1-C178R air2*Δ growth at 30°C. The *air1-C178R air2*Δ cells containing vector, TRAMP component or suppressor *2μ URA3* plasmid were spotted and grown at indicated temperatures. (**B**) *NAB3* suppresses the *air1-C178R air2*Δ thermosensitive growth at 30°C to the same degree as *NAB3* suppressor plasmid, *SUP11–3*. The *air1-C178R air2*Δ cells containing vector, *AIR1*, *TRF4*, *SUP11–3* or *NAB3 μ URA3* plasmids were spotted and grown at indicated temperatures. See also S1 Fig. (**C**) *NAB3* is not a bypass suppressor of *AIR1/2*. *NAB3* cannot improve *air1*Δ *air2*Δ growth. Two *air1*Δ *air2*Δ strains (ACY1095, ACY2036) containing *AIR2 URA3* maintenance plasmid and vector, *AIR1-GFP*, *TRF4*, *TRF5* or *NAB3* 2μ *HIS3* plasmid were spotted on control/5-FOA and grown at indicated temperatures. (**D**) *NRD1* and *SEN1* do not suppress the *air1-C178R air2*Δ thermosensitive growth at 30°C. The *air1-C178R air2*Δ cells containing vector, *TRF4*, *NAB3*, *NRD1* or *SEN1 2μ URA3* plasmid were spotted and grown at indicated temperatures. See also S1 Fig. (**E**) Exogenous Nrd1 and Sen1 proteins are expressed in *air1-C178R air2*Δ cells with Nrd1 expressed to a similar level as Nab3. Lysates of *air1-C178R air2*Δ cells expressing Myc-tagged Nab3, Nrd1 or Sen1 at 30°C were analyzed by immunoblotting to detect Myc-tagged proteins and 3-phosphoglycerate kinase (Pgk1) as a loading control. Nonadjacent lanes in the same immunoblot are separated by white space. Different immunoblots are outlined by black boxes. See also S2 Fig. (**F**) Nab3 expression does not increase the level of air1-C178R mutant protein. Lysates of *air1-C178R-TAP air2*Δ cells containing vector or expressing Trf4 or Nab3 protein grown at 25°C were analyzed by immunoblotting to detect air1-C178R-TAP protein and 3-phosphoglycerate kinase (Pgk1) as a loading control. Percentage of air1-C178R-TAP relative to Pgk1 loading control and cells containing vector alone (% air1-C178R Rel Vector) is shown below lanes and was calculated as described in Materials and Methods. Quantitation refers to specific experiment shown but is representative of multiple experiments.

To study the function of TRAMP, we exploited thermosensitive *air1-C178R air2*ΔTRAMP mutant cells, hereafter referred to as *air1/2* cells, containing an integrated C178R substitution in *AIR1* zinc knuckle 5 combined with deletion of *AIR2* [16]. The *air1/2* cells are thermosensitive, show impaired growth at 30°C, and can be suppressed by TRAMP components [16] (Fig. 1A).

To identify additional factors that facilitate TRAMP function, we performed a high-copy suppressor screen with *air1/2* cells. From 60 suppressor (SUP) plasmids that improved *air1/2* growth at 30°C, 44 plasmids contained either *AIR1* (e.g. *SUP3–2*) or *AIR2* (Fig. 1A). Four other plasmids, including *SUP11–3*, all contained a large insert including the *NAB3* gene and potently suppress *air1/2* growth at 30°C (Fig. 1A). A plasmid clone of *NAB3* suppresses *air1/2* growth at 30°C to a similar degree as suppressor *SUP11–3* (Fig. 1B), confirming that suppression is conferred by *NAB3*. *NAB3* suppression of *air1/2* growth could mean that Nab3 specifically facilitates Air1 function or more generally facilitates TRAMP function. To this point, *NAB3* can suppress the slow growth of another TRAMP mutant, *trf4*Δ, at 25°C (S1A Fig), suggesting that Nab3 facilitates TRAMP function.

As TRAMP is an exosome cofactor and Air1/2 facilitate RNA recognition, the Nab3 RNA binding protein could simply replace RNA binding function of the Air proteins in *air1/2* cells and target TRAMP/exosome to RNA substrates. This suppression mechanism predicts that *NAB3* should bypass the requirement for *AIR1/2*. However, *NAB3* cannot improve *air1*Δ*air2*Δgrowth (Fig. 1C) and thus is not a bypass suppressor of *AIR1/2* function.

To assess the specificity of *NAB3* suppression, we examined whether other RNA-binding protein genes can suppress *air1/2* growth. *NAB3* was identified in a screen for nuclear polyadenylated RNA-binding (Nab) proteins [40]. *NAB/hnRNP* genes, *NPL3/NAB1*, *HRP1/NAB4*, *PUB1 or NAB2* do not suppress *air1/2* growth (S1B Fig), indicating that *air1/2* suppression is specific to *NAB3* and that Nab3 possesses a unique function, as compared to other RNA binding proteins.

### *NRD1* and *SEN1* do not suppress *air1/2* thermosensitive cell growth

Nab3 is part of the Nrd1-Nab3-Sen1 (NNS) complex that functions in the termination and 3’-end processing/degradation of ncRNAs [19,20]. Nab3 directly binds to Nrd1 [29,30], and together, Nab3 and Nrd1 recognize terminators in ncRNAs [29]. Nab3 also binds to the RNA helicase, Sen1 [31], which is thought to unwind the RNA-DNA hybrid to terminate transcription [5]. We tested whether *NRD1* and *SEN1* could suppress *air1/2* growth. Unlike *NAB3*, *NRD1* and *SEN1* do not suppress *air1/2* growth at 30°C (Fig. 1D), even though both exogenous Nrd1 and Sen1 are expressed in *air1/2* cells (Fig. 1E). *NRD1* and *SEN1* also do not suppress *trf4*Δ slow growth (S1A Fig). Importantly, both exogenous Nrd1 and Nab3 are overexpressed by at least 8-fold relative to endogenous Nrd1 and Nab3 in *air1/2* cells (S2 Fig). As overexpression of exogenous Sen1 could not be verified at this time, it remains possible that *SEN1* does not suppress *air1/2* cells because it is not significantly overexpressed. These results indicate that *air1/2* suppression is specific to *NAB3* and highlight a functional difference between Nab3 and Nrd1.

### *NAB3* does not increase air1 mutant or Nrd1 protein levels in *air1/2* cells

Previous studies revealed that the air1-C178R mutant protein is unstable and impacts the integrity of TRAMP in *air1/2* cells [16]. Trf4 increases the air1-C178R level and suppresses *air1/2* growth, suggesting that Trf4-mediated suppression involves air1-C178R stabilization [16]. To determine whether Nab3 suppression also involves air1-C178R stabilization, we examined the air1-C178R level in *air1/2* cells that overexpress Nab3. Unlike Trf4, Nab3 expression does not increase the air1-C178R level in *air1/2* cells (Fig. 1F). Nab3 also does not appreciably alter the level of endogenous Nrd1 in *air1/2* cells (Fig. 1F).

### The Nab3 Nrd1-binding domain is not essential for *air1/2* suppression

**Fig 2:**
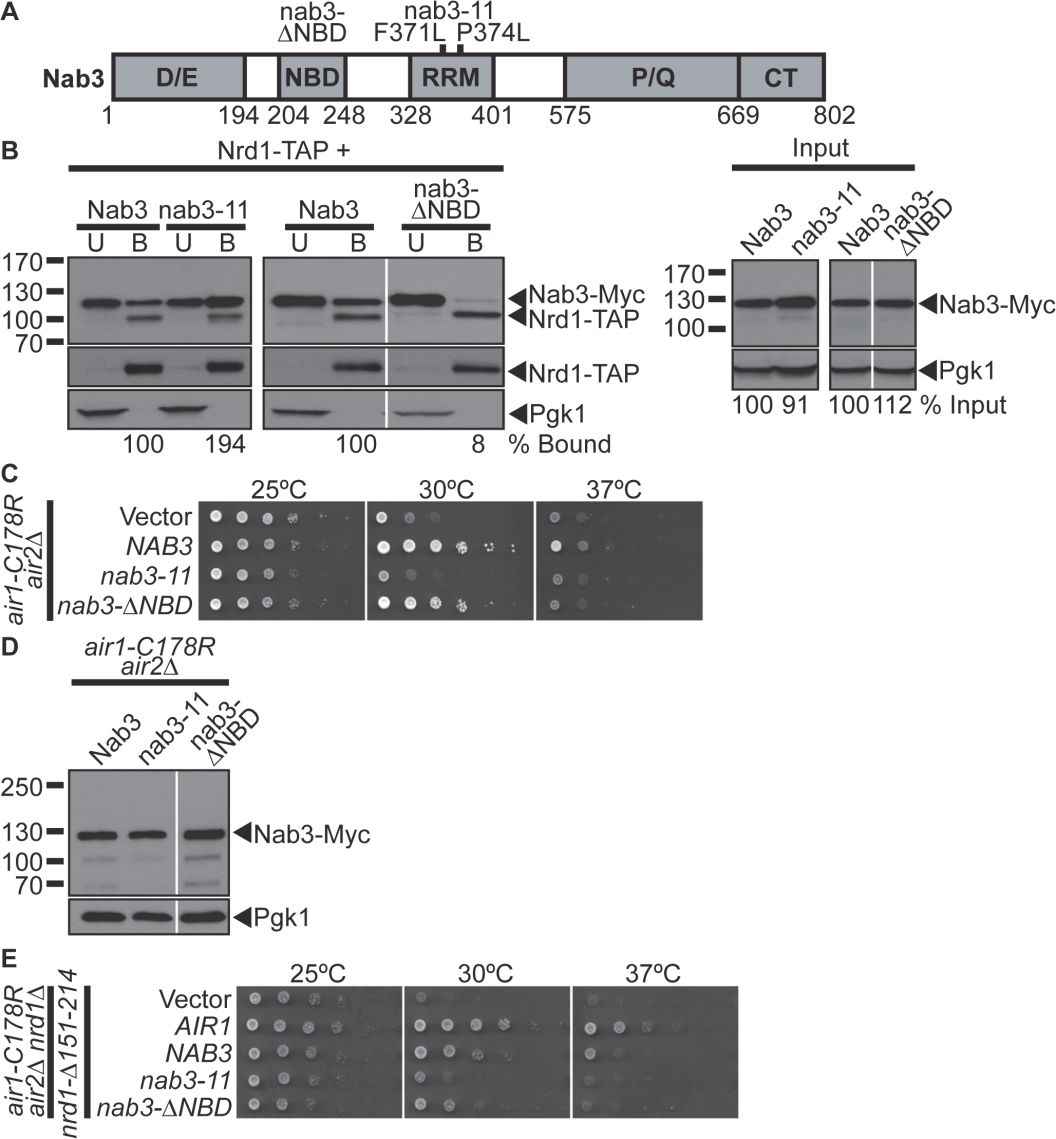
The Nab3 Nrd1-binding domain is not essential for suppression of *air1/2* thermosensitive growth. (**A**) Schematic of Nab3 depicting domain structure. Nab3 contains five domains: an Asp/Glu-rich domain (D/E; residues 1–194), a Nrd1-binding domain (NBD; 204–248), an RNA recognition motif (RRM; 328–401), a Pro/Gln-rich domain (P/Q; 575–668), and a carboxy-terminal self-association domain (CT; 669–802). Residue deletion in nab3-ΔNBD mutant and residue substitutions, F371L and P374L, in the nab3 RRM mutant, nab3–11, are depicted above. Residue positions are depicted below. See also S6 Fig for sequence. (**B**) The nab3-ΔNBD mutant protein shows greatly reduced binding to Nrd1. TAP-tagged Nrd1 was precipitated from lysates of *NRD1-TAP* cells expressing Myc-tagged Nab3, nab3–11 or nab3-ΔNBD and bound (B), unbound (U), and input fractions were analyzed by immunoblotting to detect Nab3-Myc proteins and 3-phosphoglycerate kinase (Pgk1) as a loading control. Samples were also probed with anti-Nrd1 antibody to detect Nrd1-TAP proteins. The percentage of bound Nab3 protein relative to input protein and bound wild-type Nab3 (% Bound) is shown below the bound lanes. The percentage of input Nab3 protein relative to input wild-type Nab3 protein (% Input) is shown below the input lanes. The percentages of protein were calculated as described in Materials and Methods. Quantitation refers to specific experiment shown but is representative of multiple experiments. Nonadjacent lanes in the same immunoblot are separated by white space. Different immunoblots are outlined by black boxes. The original immunoblot for the spliced immunoblot on the right is shown in S3A Fig. (**C**) The *nab3-*Δ*NBD* mutant suppresses, but the *nab3–11* RRM mutant does not suppress, *air1-C178R air2*Δ thermosensitive growth at 30°C. The *air1-C178R air2*Δ cells containing vector, *NAB3*, *nab3–11 or nab3-*Δ*NBD 2μ URA3* plasmid were spotted and grown at indicated temperatures. See also S1 and S3B Figs. (**D**) nab3-ΔNBD mutant protein is expressed in *air1-C178R air2*Δ cells to a similar level as Nab3. Lysates of *air1-C178R air2*Δ cells expressing Myc-tagged Nab3, nab3–11 or nab3-ΔNBD at 30°C were analyzed by immunoblotting detect Myc-tagged proteins and 3-phosphoglycerate kinase (Pgk1) as a loading control. Nonadjacent lanes in the same immunoblot are separated by white space. (**E**) *NAB3* and *nab3-*Δ*NBD* suppress *air1-C178R air2*Δ *nrd1-*Δ*151–214* thermosensitive growth at 30°C. The *air1-C178R air2*Δ *nrd1*Δ cells containing nrd1-Δ151–214 *CEN HIS3* plasmid and vector, *AIR1*, *NAB3*, *nab3–11*, *or nab3-*Δ*NBD 2μ URA3* plasmid were spotted and grown at indicated temperatures.

Nab3 and Nrd1 directly interact and are thought to work together to recognize RNAs for termination/processing [5,10]. However, only *NAB3* suppresses *air1/2* cells, even though Nab3 and Nrd1 are expressed at similar levels (see Fig. 1E). This result suggests that Nab3 might not require interaction with Nrd1 to suppress *air1/2* cells and could function independent of Nrd1. To address this point, we tested whether a *nab3* mutant that lacks the Nrd1-binding domain can still suppress *air1/2* cells. Previously, the Nrd1-binding domain (NBD) of Nab3 was mapped to residues 204–248 [30] (Fig. 2A). We thus generated a Nab3 deletion mutant that lacks the Nrd1-binding residues 204–248, *nab3-Δ* Δ*NBD*, to test for *air1/2* suppression. To assess whether Nab3 RNA binding is important for *air1/2* suppression, we also tested a Nab3 RNA recognition motif (RRM) mutant, *nab3–11* [22] (Fig. 2A). To confirm that nab3-ΔNBD binding to Nrd1 is impaired, we precipitated TAP-tagged Nrd1 from lysates of *NRD1-TAP* cells expressing Myc-tagged Nab3, nab3–11, and nab3-ΔNBD and analyzed the bound fractions by immunoblotting. The nab3-ΔNBD mutant protein shows greatly reduced binding to Nrd1 (8% bound) compared to wild-type Nab3 (2B and S3A Figs). In contrast, the nab3–11 RRM mutant protein shows no decrease in binding to Nrd1 (Fig. 2B).

We then tested whether the *nab3-*Δ*NBD* and *nab3–11* RRM mutants can suppress *air1/2* growth. The *nab3-*Δ*NBD* mutant suppresses *air1/2* growth similarly but not identically to *NAB3* at 30°C (Fig. 2C). The nab3-ΔNBD mutant protein is expressed at a similar level to wild-type Nab3 protein (Fig. 2D). This result suggests that the Nab3 Nrd1-binding domain is not essential for *air1/2* suppression. In contrast, the *nab3–11* mutant cannot suppress *air1/2* growth at 30°C (Fig. 2C). Notably, *nab3-*Δ*NBD*, but not *nab3–11*, also suppresses *trf4*Δ growth (S1A Fig).

To further examine whether Nab3 requires Nrd1 interaction to suppress *air1/2* cells, we also tested whether *NAB3* and *nab3-*ΔNBD can suppress *air1/2 nrd1*Δ cells that only express a nrd1-Δ151–214 variant that lacks the Nab3-binding domain. *NAB3* and *nab3-*Δ*NBD* suppress *air1/2 nrd1-*Δ*151–214* growth at 30°C (Fig. 2E). Combined, these data suggest that Nab3 does not require interaction with Nrd1, but must interact with RNA, to suppress *air1/2* growth.

**Fig 3:**
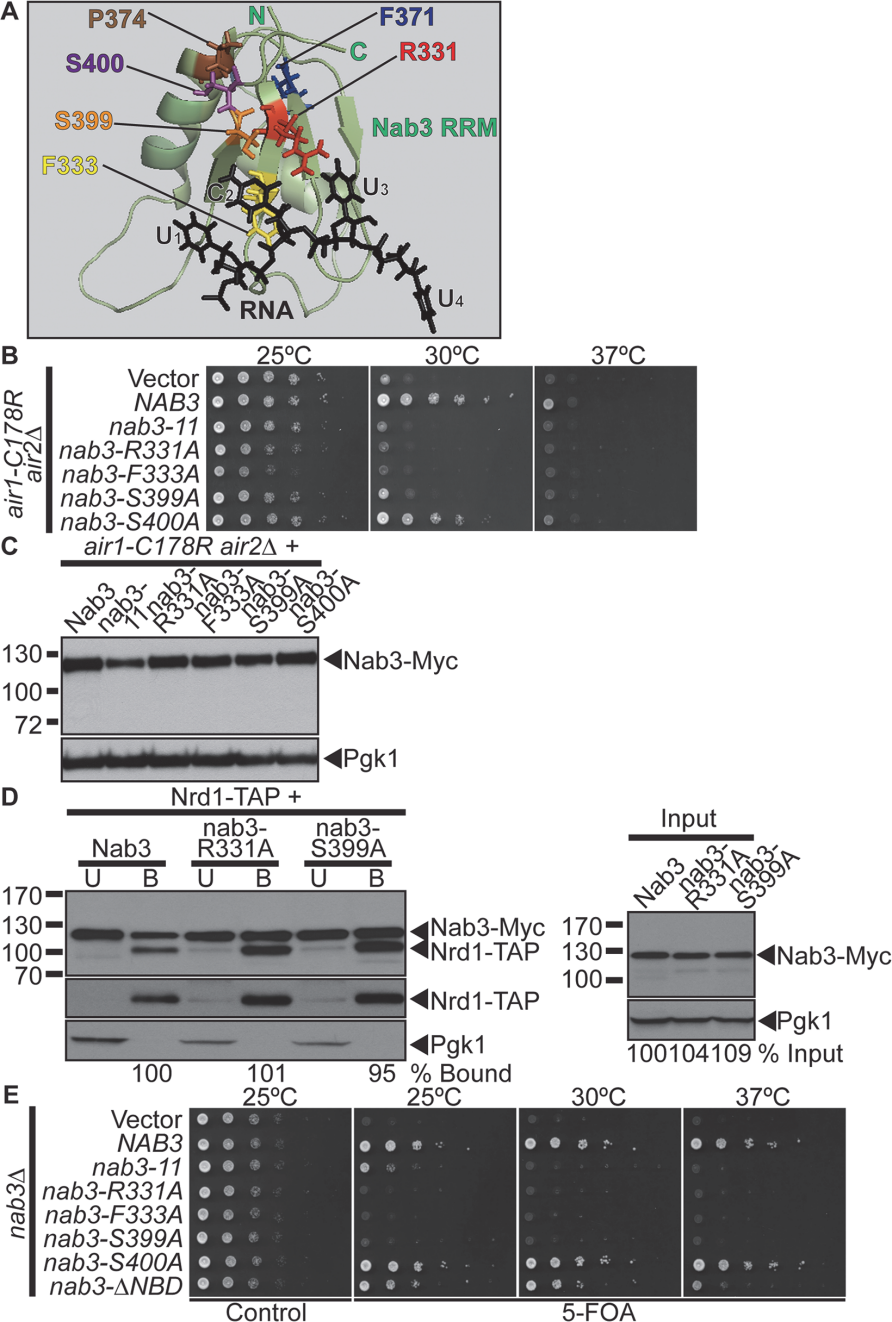
Nab3 RNA recognition motif is essential for suppression of *air1/2* thermosensitive growth. (**A**) NMR structure of Nab3 RRM in complex with UCUU RNA, which highlights key Nab3 RRM residues that contact the RNA [41]. The Nab3 RRM-RNA NMR structure (PDB ID: 2L41) shows that the Nab3 RRM forms a four-stranded β-sheet packed against two α–helices [41]. The Nab3 RRM (green; Nab3 residues 321–415) interacts with the U_1_C_2_U_3_U_4_ RNA oligonucleotide (black). Nab3 RRM β-strand residues, Arg331 (red), Phe333 (yellow), and Ser399 (orange), make specific contacts with the C_2_ nucleotide. Ser399 (orange) also contacts the U_3_ nucleotide. The Nab3 RRM residues, Phe371 (blue) and Pro374 (brown), mutated in the nab3–11 (nab3-F371L-P374L) RRM mutant, are also highlighted. The Nab3 RRM-RNA NMR structure was reproduced from the PDB file 2L41 [41] using MacPyMOL software [62] and altered and annotated using Adobe Photoshop and Illustrator CS4 (Adobe). (**B**) *nab3* RRM mutants, *nab3-R331A*, *nab3-F333A*, *nab3-S399A* do not suppress the *air1-C178R air2*Δ thermosensitive growth at 30°C. *nab3* RRM mutant, *nab3-S400A*, suppresses *air1-C178R air2*Δ thermosensitive growth at 30°C. The *air1-C178R air2*Δ cells containing vector, *NAB3* or *nab3 RRM* mutant *2μURA3* plasmid were spotted and grown at indicated temperatures. See also S1 and S2 Figs. (**C**) All nab3 RRM mutant proteins, except nab3–11, are expressed in *air1-C178R air2*Δ cells to similar levels as Nab3. Lysates of *air1-C178R air2*Δ cells expressing Myc-tagged Nab3 or nab3 RRM mutants at 30°C were analyzed by immunoblotting to detect Myc-tagged proteins and 3-phosphoglycerate kinase (Pgk1) as a loading control. (**D**) The nab3-R331A and nab3-S399A RRM mutant proteins show binding to Nrd1 similar to wild-type Nab3. TAP-tagged Nrd1 was precipitated from lysates of *NRD1-TAP* cells expressing Myc-tagged Nab3, nab3-R331A or nab3-S399A and bound (B), unbound (U), and input fractions were analyzed by immunoblotting to detect Nab3-Myc proteins, Nrd1-TAP proteins and 3-phosphoglycerate kinase (Pgk1) as a loading control. The percentage of bound Nab3 relative to input protein and bound wild-type Nab3 (% Bound) is shown below the bound lanes. The percentage of input Nab3 protein relative to input wild-type Nab3 protein (% Input) is shown below the input lanes. The percentages of protein were calculated as described in Materials and Methods. Quantitation refers to specific experiment shown but is representative of multiple experiments. The original immunoblot is shown in S3A Fig. (**E**) *nab3*Δcells expressing *nab3* RRM mutants, nab3-R331A, nab3-F333A or nab3-S399A, are not viable, but *nab3*Δ cells expressing *nab3-S400A* RRM mutant or *nab3-*Δ *NBD* mutant are viable. *nab3*Δ cells expressing *nab3–11* RRM mutant show a severe growth defect. *nab3*Δ cells containing *NAB3 URA3* maintenance plasmid and vector, *NAB3*, *nab3 RRM* mutant or *nab3-*Δ *NBD* mutant 2*μ HIS3* plasmid were spotted on control/5-FOA and grown at indicated temperatures.

As the Nab3 Nrd1-binding domain is not essential for *air1/2* suppression, we assessed whether this domain is essential for Nab3 function. We tested whether the *nab3-*Δ*NBD* mutant can function as the sole copy of the essential *NAB3* gene. The *nab3-*Δ*NBD* mutant cells are viable, but show a moderate growth defect, while *nab3–11* mutant cells exhibit a severe growth defect (Fig. 3E), indicating that the Nab3 Nrd1-binding domain and thus Nab3 interaction with Nrd1 is not essential for the cellular function of Nab3.

### The Nab3 RNA recognition motif is essential for *air1/2* suppression

The inability of the *nab3–11* RRM mutant to suppress *air1/2* growth suggests that the Nab3 RRM and RNA interaction is critical for suppression. To further analyze the importance of the Nab3 RRM in *air1/2* suppression, we took advantage of a recent NMR structure of the Nab3 RRM in complex with UCUU RNA, the Nab3 RNA binding site [41]. The Nab3 RRM bound to RNA forms a four-stranded β-sheet packed against two α-helices similar to canonical RRMs [41]. Key residues in the β-strands interact specifically with the U_1_C_2_U_3_U_4_ RNA sequence [41] (Fig. 3A). In particular, Nab3 residues Arg331, Phe333, and Ser399 make contacts with the C_2_ nucleotide and Ser399 also contacts the U_3_ nucleotide [41] (Fig. 3A). Importantly, Nab3 RRM mutants R331A and S399A show a 3–4-fold decrease in binding to RNA *in vitro* [41]. We thus generated *nab3* RRM mutants, *nab3-R331A*, *nab3-F333A*, and *nab3-S399A* and tested if these *nab3* RRM mutants can suppress *air1/2* growth. We also generated and tested the *nab3* RRM mutant, *nab3-S400A*, as the Ser400 residue neighbors the key Ser399 nucleotide-binding residue in the Nab3 RRM. The *nab3-R331A*, *nab3-F333A*, and *nab3-S399A* RRM mutants, like *nab3–11*, do not suppress *air1/2* growth at 30°C (Fig. 3B). These nab3 RRM mutants also do not suppress *trf4* Δ growth (S1A Fig). The *nab3-S400A* RRM mutant, like *NAB3*, however, suppresses *air1/2* growth at 30°C (Fig. 3B). Thus, Nab3 RRM residues, Arg331, Phe333, and Ser399, are critical for *air1/2* suppression, supporting the notion that the Nab3 RNA interaction is essential for suppression.

Critically, all the nab3 RRM mutants, except nab3–11, are expressed at similar levels to Nab3 (Fig. 3C). Expression of *nab3* RRM mutants in wild-type cells does not impair growth, showing that the inability of most *nab3* RRM mutants to suppress *air1/2* growth is not due to toxicity (S3B Fig).

The *nab3* RRM mutants could fail to suppress *air1/2* growth because mutations in the Nab3 RRM disrupt the overall folding of Nab3, rather than the Nab3 RRM alone. To test whether nab3 RRM mutants are folded correctly, we examined the interaction between nab3 RRM mutants, nab3-R331A and nab3-S399A, and Nrd1. We precipitated TAP-tagged Nrd1 from lysates of *NRD1-TAP* yeast cells expressing Myc-tagged nab3-R331, nab3-S399A or Nab3, and analyzed the bound fractions by immunoblotting. The nab3-R331A and nab3-S399A RRM mutants show binding to Nrd1 similar to wild-type Nab3 (95–101% bound; Fig. 3D). These results show that Nab3 RRM mutations do not impair interaction of Nab3 with Nrd1, indicating that overall Nab3 folding is not disrupted in these mutants.

To determine if the *nab3* RRM mutants are functional *in vivo*, we tested whether the *nab3* RRM mutants can function as the sole copy of the essential *NAB3* gene. The *nab3-R331A*, *nab3-F333A*, and *nab3-S399A* mutants are not viable, whereas *nab3-S400A* mutant has growth comparable to *NAB3* (Fig. 3E). Thus, Nab3 RNA binding is essential for Nab3 function *in vivo*.

### *NAB3* reduces *IMD2* CUT terminator readthrough product from a reporter in *air1/2* cells

**Fig 4:**
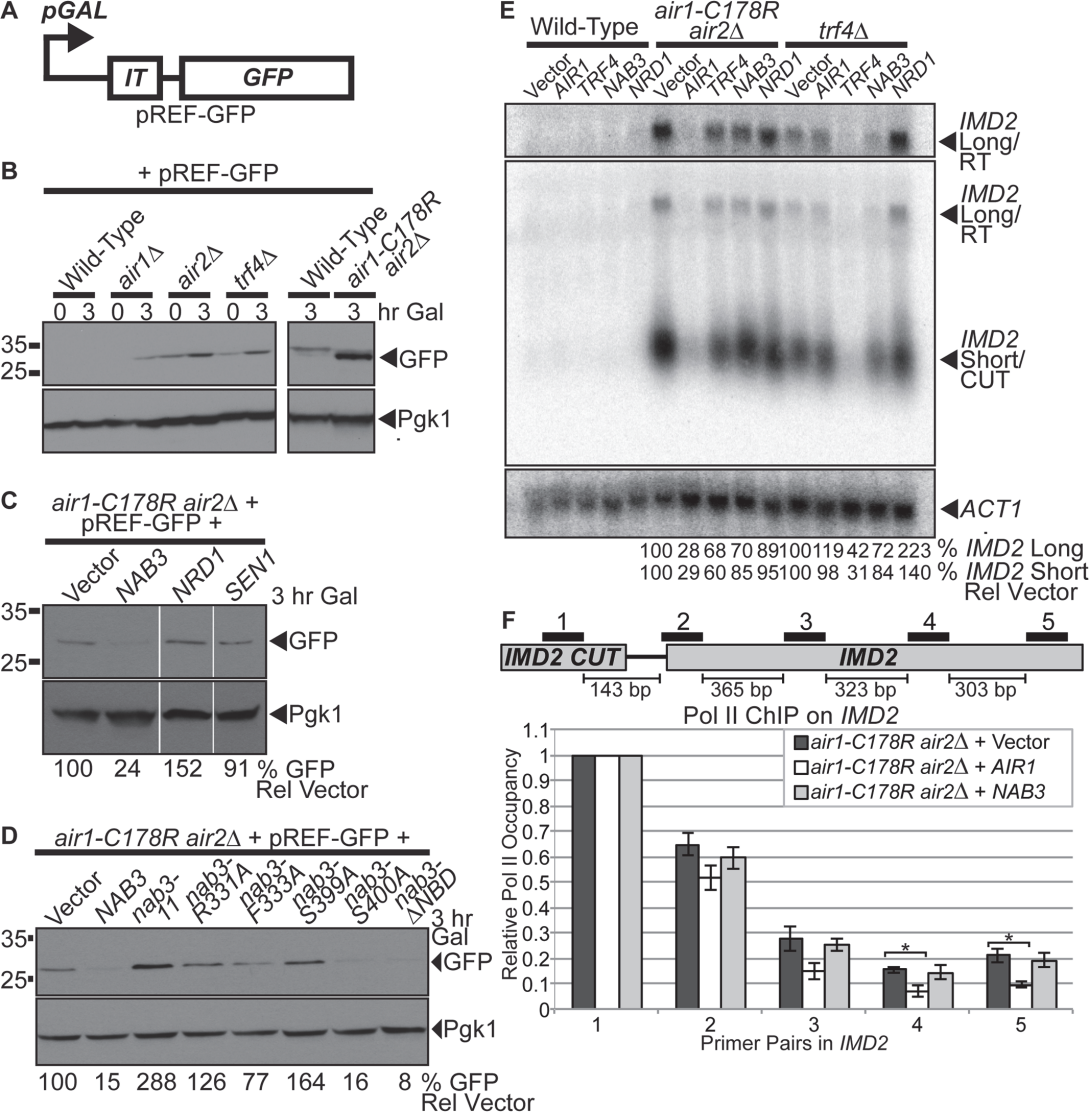
*NAB3* reduces the level of *IMD2* CUT terminator readthrough product from a reporter and native *IMD2* CUT and readthrough RNA, but *NAB3* does not significantly affect the termination of native *IMD2* CUT in *air1/2* cells. (**A**) Schematic of pREF-GFP *IMD2* CUT terminator reporter plasmid [38]. The pREF-GFP reporter contains the *IMD2* CUT intergenic terminator (*IT*) upstream of *GFP* open reading frame (*GFP*) under the control of a galactose-inducible promoter (*pGAL*). (**B**) Level of GFP readthrough product from pREF-GFP reporter is increased in *air1-C178R air2*Δ cells and other TRAMP mutants. Wild-type, *air1*Δ, *air2* **Δ**, *trf4*Δ, and *air1-C178R air2* **Δ** cells containing pREF-GFP reporter were grown in the presence of galactose (Gal) to induce to GFP expression for 3hr at 30°C and lysates were analyzed by immunoblotting to detect GFP and 3-phosphoglycerate kinase (Pgk1) as a loading control. Samples of cells just after addition of galactose (0 hour time point) were also analyzed. Different immunoblots are outlined by black boxes. (**C**) *NAB3*, but not *NRD1* or *SEN1*, decreases level of GFP readthrough product from pREF-GFP reporter in *air1-C178R air2* Δ cells. *air1-C178R air2* Δ cells containing pREF-GFP reporter plasmid and vector, *NAB3*, *NRD1* or *SEN1* were grown in the presence of galactose (Gal) to induce to GFP expression for 3hr at 30°C and lysates of cells were analyzed by immunoblotting as described previously. Nonadjacent lanes in the same immunoblot are separated by white space. (**D**) *nab3-*Δ *NBD* mutant, like *NAB3*, decreases level of GFP readthrough product from pREF-GFP reporter in *air1-C178R air2*Δ cells. *nab3-R331A*, *nab3-F333A*, and *nab3-S399A* RRM mutants do not decrease level of GFP from pREF-GFP reporter in *air1/2* cells. *air1-C178R air2*Δ cells containing pREF-GFP reporter plasmid and vector, *NAB3*, *nab3-* **Δ** *NBD* mutant or *nab3* RRM mutants were grown in the presence of galactose (Gal) to induce to GFP expression for 3hr at 30°C and lysates were analyzed by immunoblotting as described previously. The percentage of GFP relative to Pgk1 loading control and GFP in cells containing vector alone (% GFP Rel Vector) is shown below the lanes and was calculated as described in Materials and Methods. Quantitation refers to specific experiment shown but is representative of multiple experiments. (**E**) *NAB3* reduces the levels of native *IMD2* CUT and readthrough RNA in *air1-C178R air2* Δ and *trf4*Δ mutant cells. Northern blot of total RNA from wild-type, *air1-C178R air2*Δ, and *trf4*Δ cells containing vector, *AIR1*, *TRF4*, *NAB3* or *NRD1* grown at 30°C were probed with an *IMD2* CUT-specific probe. Northern blot was probed with *ACT1* probe as a loading control. The *IMD2* CUT (*IMD2* Short/CUT) and *IMD2* CUT readthrough RNA (*IMD2* Long/RT) are labeled. A longer exposure of *IMD2* CUT readthrough RNA is shown above. The percentage of *IMD2* CUT and readthrough RNA relative to *ACT1* loading control and cells containing vector alone (% *IMD2* Short Rel Vector; % *IMD2* Long Rel Vector) is shown below lanes and was calculated as described in Materials and Methods. Quantitation refers to specific experiment shown but is representative of multiple experiments. See also S4A Fig. (**F**) *NAB3* does not significantly affect Pol II occupancy downstream of the *IMD2* CUT at Primer Pair 2–5 positions in *air1-C178R air2*Δ cells relative to *air1/2* cells containing vector alone (*p*-value ≥ 0.4), suggesting that Nab3 overexpression does not significantly affect *IMD2* CUT termination in *air1/2* cells. As a control, *AIR1* significantly decreases Pol II occupancy downstream of the *IMD2* CUT in *air1-C178R air2*Δ cells at Primer Pair 4 and 5 positions compared to *air1/2* cells containing vector alone (*p*-value ≤ 0.05), indicating that Air1 significantly affects termination and suggesting that *air1/2* cells have a termination defect. Anti-Pol II ChIP was performed on *air1-C178R air2*Δ cells containing vector, *AIR1* or *NAB3* and relative Pol II occupancy was measured across *IMD2* gene by qPCR with *IMD2* Primer Pair 1–5 as described in Material and Methods. Mean RNA Pol II occupancy values from three independent experiments normalized to Primer Pair 1 within *IMD2* CUT are shown with error bars that represent standard error of the mean. Statistical significance of differences in mean Pol II occupancy values was determined using unpaired *t* test and significant differences in mean values (*p*-value ≤ 0.05) are denoted with asterisks. Schematic of *IMD2* CUT and downstream *IMD2* gene is shown with positions of *IMD2* qPCR Primer Pairs 1–5 above and base pair distances between primer pairs below. See also S4B Fig.

Nab3 and Nrd1 recognize elements in the terminators of ncRNAs/CUTs and mRNAs to facilitate termination and processing/degradation [5,20,21]. Notably, *nrd1/nab3* mutants exhibit readthrough of NNS terminators in ncRNAs and Nrd1/Nab3 binding site mutations within these NNS terminators also cause readthrough [5,8,20,21]. Moreover, *trf4* Δ cells show readthrough of RNA terminators [42,43]. To begin to assess termination of CUTs in *air1/2* cells and test the impact of *NAB3* on CUT termination, we employed a pREF-GFP reporter for CUT terminator readthrough [38]. The pREF-GFP reporter contains a galactose-inducible promoter and the *IMD2* CUT intergenic terminator (IT) upstream of *GFP* [38] (Fig. 4A). In wild-type cells, upon galactose induction, little GFP is observed as transcription is efficiently terminated at the terminator before *GFP*. In termination defective cells, GFP is produced as transcription is not terminated at the terminator and readthrough to *GFP* occurs. We galactose-induced the pREF-GFP reporter in wild-type, *air1/2*, and other TRAMP mutants and examined the GFP level in the cells by immunoblotting. GFP expression is increased in *air1/2* and TRAMP mutants containing pREF-GFP (Fig. 4B).

Having established that *air1/2* cells express GFP from pREF-GFP, suggesting readthrough of the *IMD2* CUT terminator and/or impaired degradation of the readthrough RNA, we wished to determine if *NAB3* impacts the GFP level in *air1/2* cells containing pREF-GFP. *NAB3* greatly reduces the GFP level (24% GFP), *NRD1* increases the GFP level (152% GFP), and *SEN1* does not affect the GFP level (91% GFP) in *air1/2* cells (Fig. 4C). These data suggest that *NAB3* reduces the readthrough of the *IMD2* CUT terminator and/or increases the degradation of the *IMD2* readthrough RNA in *air1/2* cells, suggesting that *NAB3* can improve the termination/degradation of CUTs in *air1/2* cells.

To determine whether the *NAB3* effect on *IMD2* terminator readthrough and/or *IMD2* readthrough RNA degradation in *air1/2* cells requires Nab3 interaction with RNA or Nrd1, we tested if the *nab3* RRM mutants and *nab3–204–248* NBD mutant affect the GFP level. Most *nab3* RRM mutants do not reduce the GFP level (126–288% GFP), but the *nab3-*Δ*NBD* mutant greatly decreases the GFP level (8% GFP) in *air1/2* cells (Fig. 4D). These data suggest that the *NAB3*-mediated decrease in *IMD2* CUT terminator readthrough and/or increase in *IMD2* readthrough RNA degradation in *air1/2* cells requires the Nab3 RRM, but does not require Nrd1 interaction.

### *NAB3* decreases the levels of native *IMD2* CUT and readthrough RNA in *air1/2* and *trf4Δ* cells

*NAB3* reduction of the GFP level from the pREF-GFP reporter in *air1/2* cells suggests that Nab3 decreases *IMD2* CUT readthrough, but the decrease in GFP level could also result from downstream effects of Nab3 on RNA processing/degradation or translation. To test if *NAB3* affects the native *IMD2* CUT in *air1/2* or *trf4*Δ cells, we probed a Northern blot of total RNA from *air1/2*, *trf4*Δ or wild-type cells containing vector, *AIR1*, *TRF4*, *NAB3* or *NRD1*, with an *IMD2* CUT-specific probe. The level of the short *IMD2* CUT is greatly increased in *air1/2* and *trf4*Δ cells with vector alone, compared to wild-type cells with vector alone, as expected for TRAMP mutants that impair exosome degradation of CUTs (Fig. 4E). Importantly, the level of a long *IMD2* CUT readthrough RNA is also increased in *air1/2* and *trf4*Δ cells with vector alone, relative to wild-type cells, consistent with the increased GFP from pREF-GFP in these cells and supporting the notion that TRAMP mutants also have termination defects (Fig. 4E). *AIR1* greatly reduces to 28–29% and *TRF4* partially reduces to 60–68% the levels of the *IMD2* CUT and readthrough RNA in *air1/2* cells, relative to cells containing vector alone, consistent with *AIR1* and *TRF4* reactivating TRAMP degradation/termination (Fig. 4E). *NAB3* reduces to 85% the level of the *IMD2* CUT, whereas *NRD1* reduces to 95% the level of the *IMD2* CUT, in *air1/2* cells (Fig. 4E). Moreover, *NAB3* decreases to 70% the *IMD2* CUT readthrough RNA, whereas *NRD1* decreases to 89% the readthrough RNA, in *air1/2* cells (Fig. 4E). In addition, *NAB3* reduces to 72–84% the level of *IMD2* CUT and readthrough RNA, but *NRD1* does not decrease the level of the *IMD2* CUT and readthrough RNA in *trf4*Δ cells (Fig. 4E). These data indicate that *NAB3* can reduce both the *IMD2* CUT and readthrough RNA in *air1/2* cells and does so to a greater extent than *NRD1*, suggesting that Nab3 affects the degradation/termination of the *IMD2* CUT and readthrough RNA to a larger degree than Nrd1. *NAB3* suppression of TRAMP mutant growth therefore correlates with *NAB3* reduction of *IMD2* CUT and readthrough RNA in TRAMP mutant cells.

To determine if the *NAB3* decrease of the *IMD2* CUT and readthrough RNA in *air1/2* cells is dependent on Nab3 interaction with RNA or Nrd1, we probed a Northern blot of total RNA from *air1/2* cells containing vector, *NAB3*, *nab3* RRM mutants or *nab3-*Δ *NBD* mutant with an *IMD2* CUT-specific probe. *nab3-*Δ *NBD* decreases the level of *IMD2* CUT and readthrough RNA similar to *NAB3* in *air1/2* cells (S4A Fig). In contrast, the *nab3* RRM mutants, *nab3–11*, *nab3-R331A*, and *nab3-S399A*, decrease the level of the *IMD2* CUT and readthrough RNA to a lesser degree than *NAB3* (S4A Fig). These data support the idea that Nab3 requires RNA interaction, but does not require Nrd1 binding, to terminate/degrade and decrease the *IMD2* CUT readthrough RNA.

### *NAB3* does not significantly affect termination of native *IMD2* CUT in *air1/2* cells

The Nab3-mediated decrease in the level of the *IMD2* CUT readthrough RNA detected in *air1/2* cells could be due to increased termination and/or increased degradation. To assess whether Nab3 affects the termination of the native *IMD2* CUT in *air1/2* cells, we examined RNA Pol II occupancy on the *IMD2* gene in *air1/2* cells containing vector, *AIR1* or *NAB3* by Pol II ChIP. We employed five primer pairs to examine Pol II occupancy and normalized all data back to Primer Pair 1 located within the *IMD2* CUT (Fig. 4F). Given that the chromatin was sheared to a size ranging from 300–500 base pairs and the distances between the primer pairs (Fig. 4F), we expected to detect differences in Pol II occupancy most readily with Primer Pair 4 and 5. The *air1/2* cells expressing *AIR1* show a statistically significant decrease in Pol II occupancy downstream of the *IMD2* CUT at Primer Pair 4 and 5 positions relative to the *air1/2* cells containing vector alone (*p*-value ≤ 0.05; Fig. 4F). In contrast, the *air1/2* cells expressing *NAB3* show no statistically significant change in Pol II occupancy downstream of the *IMD2* CUT at Primer Pair 2–5 positions relative to *air1/2* cells containing vector alone (*p*-value ≥ 0.4; Fig. 4F). These data suggest that overexpression of Nab3 does not significantly affect termination of the *IMD2* CUT in *air1/2* cells. To extend this analysis, we also performed Pol II ChIP on the native *snR13* snoRNA gene, which contains a well-characterized NNS-dependent terminator [20,21,44], in *air1/2* cells containing vector or *NAB3*. As described for the *IMD2* CUT, *air1/2* cells expressing *NAB3* do not exhibit a statistically significant change in Pol II occupancy downstream of *snR13* compared to *air1/2* cells containing vector alone (*p*-value ≥ 0.3; S4B Fig), suggesting overexpression of Nab3 does not significantly affect termination of the *snR13* gene in *air1/2* cells. These results indicate that Nab3 overexpression does not have a significant effect on the termination of the *IMD2* CUT, suggesting that Nab3 suppression of *air1/2* cells and reduction of *IMD2* CUT readthrough RNA predominantly involves Nab3 rescue of degradation.

### *NAB3* suppression of *air1/2* and reduction of *IMD2* CUT and readthrough RNA is dependent on *RRP6*

**Fig 5:**
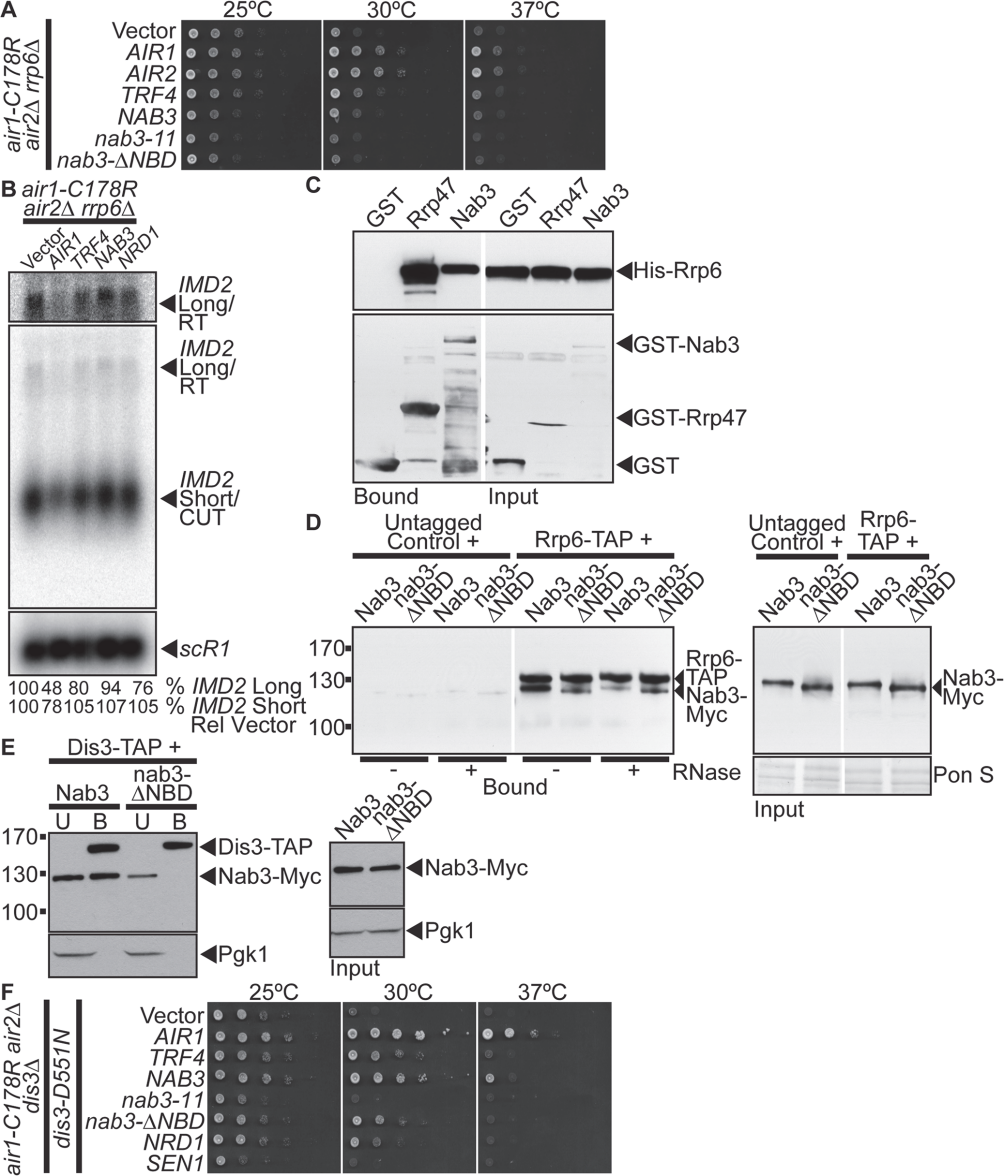
*NAB3* suppression of *air1/2* thermosensitive growth and reduction of *IMD2* CUT and readthrough RNA in *air1/2* cells is dependent on *RRP6* and Nab3 interacts with Rrp6 independent of Nrd1. (**A**) *NAB3* and *nab3-*Δ *NBD* mutant do not suppress the thermosensitive growth of *air1-C178R air2*Δ *rrp6*Δ cells that contain a deletion of *RRP6* at 30°C. *air1-C178R air2*Δ *rrp6*Δ cells containing vector, *AIR1*, *AIR2*, *TRF4*, *NAB3*, *nab3–11*, *nab3-*Δ *NBD* or *NRD1 2 μ URA3* plasmid were spotted and grown at indicated temperatures. See also S5A Fig. (**B**) *NAB3* does not decrease the levels of native *IMD2* CUT and only weakly decreases the *IMD2* CUT readthrough RNA in *air1-C178R air2*Δ *rrp6*Δ cells. Northern blot of total RNA from *air1-C178R air2*Δ *rrp6*Δ cells expressing vector, *AIR1*, *TRF4*, *NAB3* or *NRD1* grown at 30°C were probed with an *IMD2* CUT-specific probe. Northern blot was probed with *scR1* probe as a loading control. The *IMD2* CUT (*IMD2* Short/CUT) and *IMD2* CUT readthrough RNA (*IMD2* Long/RT) are labeled. A longer exposure of *IMD2* CUT readthrough RNA is shown above. The percentage of *IMD2* CUT and readthrough RNA relative to *sCR1* loading control and cells containing vector alone (% *IMD2* Short Rel Vector; % *IMD2* Long Rel Vector) is shown below lanes and was calculated as described in Materials and Methods. Quantitation refers to specific experiment shown but is representative of multiple experiments. **(C)** Nab3 directly interacts with Rrp6 *in vitro*. Recombinant GST-Nab3, GST-Rrp47 or GST was incubated with recombinant His-tagged Rrp6 and glutathione Sepharose beads and bound and input fractions were analyzed by SDS-PAGE and immunoblotting to detect His-Rrp6 and GST fusion proteins. Nonadjacent lanes in the same immunoblot are separated by white space. (**D**) nab3-ΔNBD mutant protein binds to Rrp6 in an RNA-independent manner. TAP-tagged Rrp6 was precipitated from lysates of *RRP6-TAP* or untagged control cells expressing Myc-tagged Nab3 or nab3-ΔNBD in the absence or presence of RNase A. Bound and input fractions were analyzed by immunoblotting to detect Nab3-Myc proteins and membrane was stained with Ponceau S (Pon S) stain to detect total protein as a loading control. Nonadjacent lanes in the same immunoblot are separated by white space. Different immunoblots are outlined by black boxes. See also S5B Fig. (**E**) nab3-ΔNBD mutant protein, unlike wild-type Nab3, does not bind to Dis3. TAP-tagged Dis3 was precipitated from lysates of *DIS3-TAP* cells expressing Myc-tagged Nab3 or nab3-ΔNBD and bound (B), unbound (U), and input fractions were analyzed by immunoblotting to detect Nab3-Myc proteins and 3-phosphoglycerate kinase (Pgk1) as a loading control. Different immunoblots are outlined by black boxes. (**F**) *NAB3* and *nab3-*Δ*NBD* mutant suppress the thermosensitive growth of *air1-C178R air2*Δ*dis3*Δcells expressing a catalytically inactive dis3 mutant, dis3-D551N. *air1-C178R air2*Δ*dis3*Δcells containing *dis3-D551N* and vector, *AIR1*, *TRF4*, *NAB3*, *nab3–11*, *nab3-*Δ*NBD*, *NRD1* or *SEN1 2 μ URA3* plasmid were spotted and grown at indicated temperatures.

The TRAMP and NNS complexes are intimately linked to the catalytic exosome subunit, Rrp6, which is critical for processing/degrading ncRNAs/CUTs [10]. In particular, TRAMP stimulates Rrp6-mediated degradation of RNA *in vitro* [45], Nrd1 co-precipitates with Rrp6 [19], and the *nab3–11* RRM mutant exhibits a negative genetic interaction with *RRP6* [46]. We therefore examined whether *NAB3* suppression of *air1/2* cells depends on *RRP6*. We deleted *RRP6* from the *air1/2* cells and tested if *NAB3* could suppress the growth of *air1/2 rrp6*Δ cells at 30°C. We also tested if *AIR1*, *AIR2*, *TRF4*, *nab3–11* RRM mutant or *nab3-*Δ *NBD* mutant could suppress the *air1/2 rrp6*Δ mutant. *AIR1*, *AIR2*, and *TRF4* suppress *air1/2 rrp6*Δ growth at 30°C (Fig. 5A). However, strikingly, both *NAB3* and *nab3-*Δ *NBD* do not suppress *air1/2 rrp6*Δ growth at 30°C (Fig. 5A). *nab3–11* also does not suppress the *air1/2 rrp6*Δ cells (Fig. 5A). These results indicate that *NAB3* suppression of *air1/2* growth requires *RRP6*.

To ascertain if the catalytic activity of Rrp6 is required for *NAB3* suppression of the *air1/2* growth, we tested whether *NAB3* can suppress *air1/2 rrp6*Δcells expressing the catalytically inactive mutant of Rrp6, rrp6-D238A [47]. *NAB3* does not suppress *air1/2 rrp6*Δ cells containing *rrp6-D238A* indicating that *NAB3* suppression is dependent on the catalytic activity of Rrp6 (S5A Fig).

As *NAB3* reduces the levels of *IMD2* CUT and CUT readthrough RNA in *air1/2* cells, we determined if *NAB3* reduction of these RNA levels is dependent on *RRP6*. We probed a Northern blot of total RNA from *air1/2 rrp6*Δ cells containing vector, *NAB3*, *AIR1*, *TRF4* or *NRD1* with an *IMD2* CUT-specific probe. *AIR1* decreases to 48–78% the levels of the *IMD2* CUT and readthrough RNA in *air1/2 rrp6*Δ cells (Fig. 5B). In contrast, *NAB3* does not decrease (107%) the level of the *IMD2* CUT and only weakly decreases to 94% the *IMD2* readthrough RNA in *air1/2 rrp6*Δ cells (Fig. 5B). These data indicate that *NAB3* reduction of the *IMD2* CUT and readthrough RNA depends on *RRP6*, suggesting Nab3 enhanced degradation of the *IMD2* CUT requires Rrp6.

### Nab3 interacts with Rrp6 independent of Nrd1

The requirement of *RRP6* for *NAB3* suppression of *air1/2* growth and reduction of the *IMD2* CUT and readthrough RNA suggests that Nab3 may physically interact with Rrp6. Nrd1 coprecipitates with Rrp6 [19], raising the possibility, given the Nrd1-Nab3 interaction, that Nab3 could interact with Rrp6. To test if Nab3 directly interacts with Rrp6, we examined the binding of recombinant GST-tagged full-length Nab3 to His-tagged full-length Rrp6 in an *in vitro* protein binding assay. As controls, we also tested the binding of GST and GST-tagged Rrp47, an exosome cofactor known to directly interact with Rrp6 [48], to His-Rrp6. GST-Nab3 binds to His-Rrp6 but not as strongly as GST-Rrp47 binds to His-Rrp6 (Fig. 5C). This result shows that Nab3 directly interacts with Rrp6 and indicates that Nab3 can bind Rrp6 independent of Nrd1.

To determine if Nab3 can bind to Rrp6 independent of interactions with Nrd1 or RNA in yeast, we precipitated TAP-tagged Rrp6 from lysates of *RRP6-TAP* yeast cells expressing Myc-tagged Nab3 or nab3-ΔNBD mutant in the absence or presence of RNase A, and analyzed the bound fractions by immunoblotting. In the absence of RNase, binding of nab3-Δ NBD to Rrp6 is reduced but not abolished compared to Nab3 (Fig. 5D). In the presence of RNase, binding of nab3-Δ NBD to Rrp6 is similar to Nab3 (Fig. 5D). Importantly, the Nab3-Rrp6 interaction is reduced but not abolished by RNase-treatment and the nab3-NBD-Rrp6 interaction is not decreased by RNase-treatment (Fig. 5D). These results indicate that a proportion of Nab3 can interact with Rrp6 in the absence of the Nrd1-binding domain and RNA, suggesting that Nab3 can bind to Rrp6 independent of Nrd1. In further support, we performed a reverse Nab3-Rrp6 coprecipitation and found that Rrp6 binds to both Nab3 and a nab3-Δ1–248 NBD mutant (S5B Fig).

As Nab3 interacts with the catalytic exosome subunit, Rrp6, we determined if Nab3 can also interact with the other main catalytic subunit of the core exosome, Dis3/Rrp44. Notably, Nrd1 coprecipitates with Dis3 [19]. To assess interaction between Nab3 and Dis3, we precipitated TAP-tagged Dis3 from cells expressing Myc-tagged Nab3 and examined the bound fraction by immunoblotting. Nab3 co-purifies with Dis3 (Fig. 5E). To assess whether Nab3 can interact with Dis3 independent of Nrd1, we precipitated TAP-tagged Dis3 from cells expressing the nab3-ΔNBD mutant and assayed for co-purification of Nab3. Strikingly, unlike Nab3, the nab3-ΔNBD mutant does not bind to Dis3 (Fig. 5E). These results indicate that the Nab3 interaction with Dis3 is dependent upon the Nab3 Nrd1-binding domain and therefore likely interaction with Nrd1.

Given that *NAB3* suppression of *air1/2* cells is dependent on Rrp6 catalytic activity, we tested whether *NAB3* suppression of *air1/2* cells also requires Dis3 catalytic activity. *NAB3* and the *nab3-*Δ*NBD* mutant suppress the thermosensitive growth of *air1/2 dis3*Δcells that express an exonucleolytically inactive mutant of Dis3, dis3-D551N [49] (Fig. 5F). This result indicates that *NAB3* suppression of *air1/2* cells is not dependent on Dis3 exonucleolytic activity.

### Human RALY protein suppresses the thermosensitive growth of *air1/2* cells

**Fig 6:**
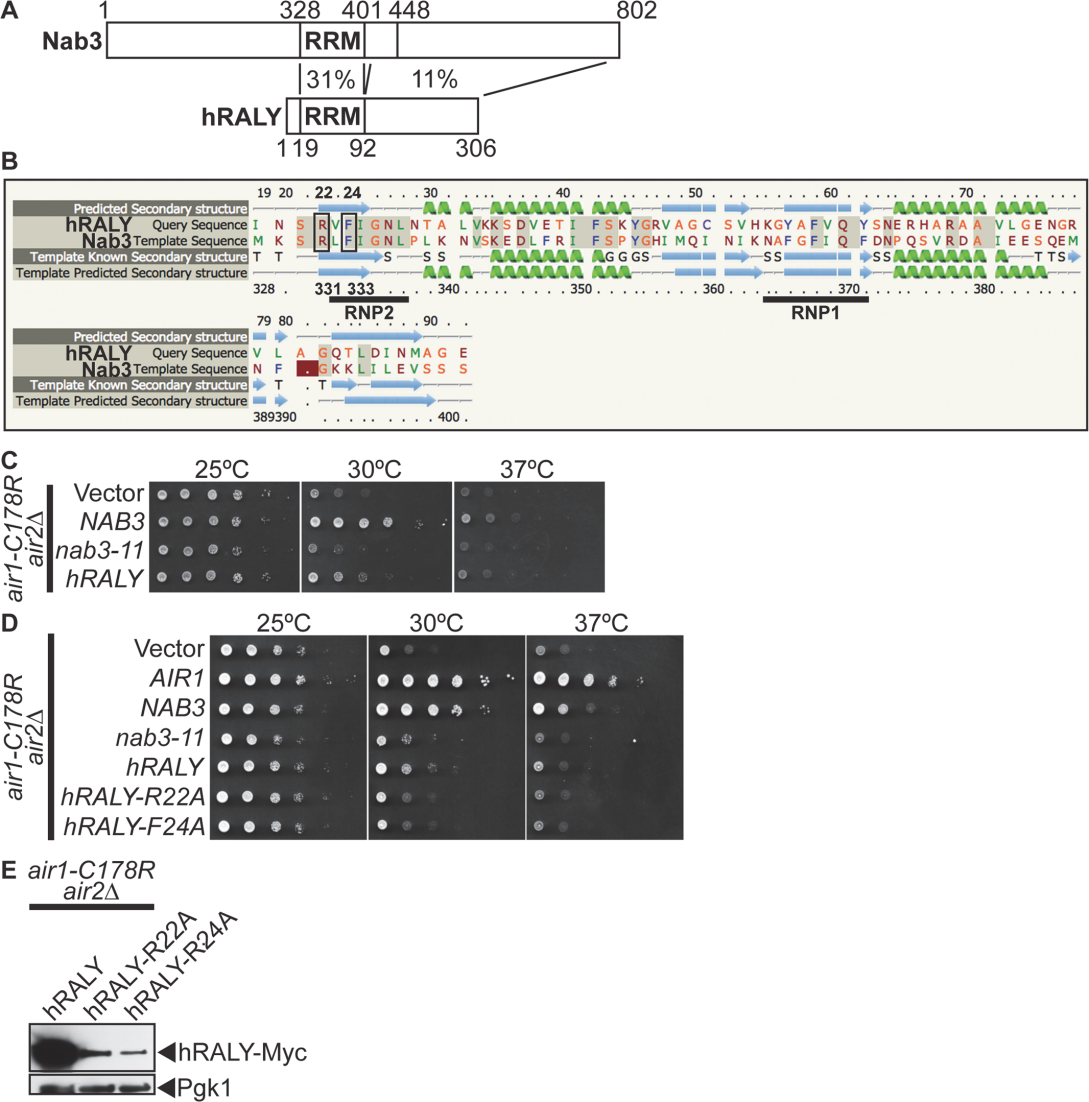
Human RALY RNA binding protein suppresses *air1/2* thermosensitive growth. (**A**) Schematic comparison of Nab3 and human RALY (hRALY) Isoform 1 (GenBank accession number Q9UKM9) RNA binding protein showing percentage sequence identity between RNA recognition domain (RRM) and C-terminal domains. Residue positions are depicted above and below proteins. See also S6 Fig. **(B)** Alignment of hRALY and Nab3 RRMs showing that the hRALY RRM has 31% identity (identical residues shaded in gray) and a similar predicted β1α1β2β3 α2β4 secondary structure (99.8% confidence; α-helices in green; β-sheets in blue) to the Nab3 RRM. Nab3 RRM RNA-binding residues R331 and F333 that are conserved in the hRALY RRM as R22 and F24 are boxed. RRM consensus motifs RNP1 and RNP2 are underlined. Alignment and secondary structure prediction of hRALY RRM based on Nab3 RRM crystal structure (PDB ID: 2XNQ [63]) was generated by Phyre2 server [64] (**C**) *hRALY* suppresses the *air1-C178R air2*Δ thermosensitive growth at 30°C. The *air1-C178R air2*Δ cells containing vector, *NAB3*, *nab3–11* or *hRALY 2μ URA3* plasmid were spotted and grown at indicated temperatures. **(D)** *hRALY* RRM mutants *hRALY-R22A* and *hRALY-F24A* do not suppress the *air1-C178R air2*Δ thermosensitive growth at 30°C. The *air1-C178R air2*Δ cells containing vector, *AIR1*, *NAB3*, *nab3–11*, *hRALY*, *hRALY-R22A* or *hRALY-F24A 2μ URA3* plasmid were spotted and grown at indicated temperatures. **(E)** hRALY-R22A and hRALY-F24A RRM mutant proteins are expressed in *air1-C178R air2* Δ cells but not to the same level as hRALY. Lysates of *air1-C178R air2∆* cells expressing Myc-tagged hRALY, hRALY-R22A or hRALY-F24A at 30°C were analyzed by immunoblotting to detect Myc-tagged proteins and 3-phosphoglycerate kinase (Pgk1) as a loading control. See also S7A–S7C Fig.

To address the question of functional conservation of Nab3, we performed a BLAST search with the Nab3 RRM and identified the human RALY (hRALY) protein that contains an RRM and C-terminal domain with homology to Nab3 (Figs. 6A and S6). The hRALY RRM shares 31% identity (23/74 residues) and a similar predicted β1α1β2β3α2β4 secondary structure with the Nab3 RRM (Fig. 6B). Moreover, the Nab3 RRM RNA-interacting residues Arg331 and Phe333 are conserved in the hRALY RRM as Arg22 and Phe24 (Fig. 6B). Importantly, hRALY can suppress the growth defect of *air1/2* TRAMP mutant cells (Fig. 6C). In contrast, hRALY RRM mutants, hRALY-R22A and hRALY F24A, cannot suppress the growth of *air1/2* cells (Fig. 6D). However, the hRALY RRM mutants are expressed to lower levels than the wild-type hRALY protein (Fig. 6E), leaving open the caveat that the inability of hRALY RRM mutants to suppress *air1/2* cells could be due to low expression levels and not to impairment of the RRM/RNA binding function of hRALY. At this time, it is therefore not possible to definitively conclude whether RNA binding by hRALY is required for suppression. Although Nab3 and hRALY share similar RRMs, the Nab3 RRM alone is not sufficient to suppress *air1/2* cells. A Nab3 truncation mutant nab3–1–448, which lacks the C-terminal 354 amino acids, but retains the intact RRM, also does not suppress *air1/2* cells (S7A Fig), even though the truncated protein is expressed (S7B Fig) and properly localized to the nucleus (S7C Fig). The C-terminal region of hRALY with homology to the C-terminal domain of Nab3 could therefore contribute to hRALY suppression of *air1/2* cells.

## Discussion

**Fig 7:**
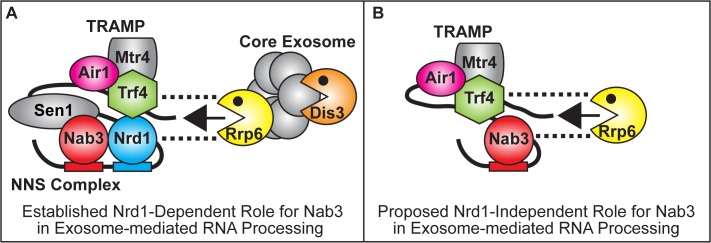
Model for Nrd1-dependent and Nrd1-independent roles of Nab3 in facilitating TRAMP function in the recruitment of the exosome for RNA processing/degradation. **(A)** In the established Nrd1-dependent model, Nab3 works in partnership with Nrd1 in the NNS complex to bind to ncRNA terminators, interact with TRAMP and recruit Rrp6 and the core exosome to terminate and process/degrade ncRNAs [5,19,29,30] (**B**) In the proposed Nrd1-independent model, Nab3 facilitates TRAMP by recruitment of Rrp6 to ncRNAs for processing/degradation independent of Nrd1. Dashed lines reflect direct physical interactions between Nrd1, Trf4 or Nab3 and Rrp6 [32; This Study].

Non-coding RNAs (ncRNAs) play key roles in gene regulation and disease [1–4]. Understanding how the TRAMP and NNS complex exosome cofactors and the exosome coordinate the processing of ncRNAs is therefore critically important. Here, we find that the Nab3 RNA-binding protein but not Nrd1 of the NNS complex facilitates the function of TRAMP in ncRNA processing/degradation, suggesting a key functional difference between Nab3 and Nrd1. Nab3 suppresses the thermosensitive growth and reduces ncRNA levels of TRAMP mutants independent of Nrd1 interaction. Moreover, Nab3 improvement of TRAMP mutant growth is dependent on the catalytic activity of the nuclear exosome subunit Rrp6 but not the core exosome subunit Dis3. In addition, Nab3 directly binds to Rrp6 and Nab3 coprecipitates with Rrp6 independent of Nrd1 interaction. In the established Nrd1-dependent model for Nab3 function, Nab3 works together with Nrd1 in the NNS complex to recognize ncRNA terminators, interact with TRAMP, and recruit Rrp6/core exosome to terminate and process/degrade ncRNAs (Fig. 7A) [5,19,29,30]. The data presented here suggest a Nrd1-independent model for Nab3 function in which Nab3 facilitates TRAMP by recruitment of Rrp6 to ncRNAs for processing/degradation independent of Nrd1 (Fig. 7B). This model raises the possibility that Nrd1 and Nab3 can function independently to recruit the exosome to ncRNA targets, allowing combinatorial flexibility in processing of RNAs. We also find that the human RALY protein that shares homology with Nab3 improves the function of TRAMP mutants.

The NNS complex and TRAMP exosome cofactors facilitate termination of ncRNAs and recruit/stimulate the exosome to process/degrade these ncRNAs (Fig. 7A). However, the mechanisms and interactions employed by the NNS and TRAMP complexes to recruit Rrp6 and the core exosome are not fully characterized. Nrd1 coprecipitates with Rrp6, the core exosome, and TRAMP and stimulates the activity of purified exosome *in vitro* [19]. Moreover, Nrd1 directly interacts weakly with Rrp6 and strongly with Trf4 [32]. Putative human TRAMP components (hTRF4–2, ZCCHC7, hMTR4) also coprecipitate strongly with hRRP6 [18]. In addition, Trf4 also directly interacts strongly with Rrp6 and TRAMP stimulates the activity of Rrp6 *in vitro* [32,45]. Both Nrd1 and TRAMP therefore interact with Rrp6. We now find that Nab3 directly interacts with Rrp6 and a nab3 mutant that lacks the Nrd1-binding domain coprecipitates with Rrp6 but not with Dis3/core exosome. Nab3 and Nrd1 of the NNS complex and TRAMP may thus independently recruit Rrp6 to ncRNA targets, providing flexibility in processing/degradation. The NNS complex and TRAMP may also interact together via Nrd1-Trf4 interaction to enhance recruitment of Rrp6 and the core exosome to ncRNA targets for rapid processing/degradation.

To better understand TRAMP function, we utilized *air1/2* TRAMP mutant cells [16]. In *air1/2* cells, the air1-ZnK5 protein shows reduced stability and decreased binding to Trf4 that leads to reduced integrity of TRAMP [16]. The primary defect in *air1/2* cells, as in other TRAMP mutants, is reduced TRAMP integrity/function that leads to decreased recruitment/stimulation of Rrp6/core exosome and thus defective processing/degradation of ncRNAs and some mRNAs [12,13,16]. Impaired TRAMP function in *trf4*Δ mutant cells also causes terminator readthrough of snoRNA, CUT, and some mRNA genes [13,42,43], suggesting TRAMP plays a role in transcription termination. In support, we find that Air1 significantly reduces RNA Pol II occupancy downstream of the *IMD2* CUT in *air1/2* cells, indicating that Air1 and TRAMP affect termination and suggesting that *air1/2* cells have a termination defect. Exactly how TRAMP affects transcription termination is an important question for future study. Impaired growth of TRAMP mutant cells is thus correlated with undegraded and/or non-terminated RNAs. Notably, polyadenylation defective TRAMP mutant cells are viable [13], indicating that polyadenylation is not the essential function of TRAMP and suggesting that exosome recruitment and other activities, such as termination, are the more vital functions of TRAMP.

In this study, we identified *NAB3* as a potent suppressor of the impaired growth of *air1/2* and *trf4*Δ TRAMP mutant cells, linking Nab3 of the NNS complex to TRAMP function. Surprisingly, *NRD1* does not suppress *air1/2* cells and a *nab3* mutant that lacks the Nrd1-binding domain still suppresses *air1/2* cells, suggesting Nab3 harbors a Nrd1-independent function that it shares with TRAMP. As *NAB3* suppression and decrease of *IMD2* CUT RNA in *air1/2* cells is dependent on Rrp6, and Nab3 directly interacts with Rrp6, we suggest that the Nab3 mechanism of suppression of *air1/2* cells involves Nab3 recruitment of Rrp6 to target ncRNAs for processing/degradation (Fig. 7B). Given that TRAMP recruits/stimulates Rrp6, this Nab3 suppression mechanism would seem logical. In support of a Nab3 role in RNA degradation, mutation of a Nab3 binding site in the *IMD2* CUT terminator leads to an increase in *IMD2* CUT RNA [28]. Importantly, *NAB3* does not significantly affect RNA Pol II occupancy downstream of the *IMD2* CUT or the *snR13* gene in *air1/2* cells, suggesting that the Nab3 suppression mechanism does not involve Nab3 improvement of termination. Conceivably, the Nab3 suppression mechanism could also involve an as yet uncharacterized function of Nab3 in TRAMP activity or the ability of Nab3 to reduce the cellular requirement for TRAMP.

The finding that *NAB3*, but not *NRD1*, suppresses the growth of TRAMP mutants is surprising, given that Nrd1 directly interacts with Nab3, Trf4, and Rrp6 [29,32]. As the Nab3 RRM is essential for suppression of TRAMP mutants and Nab3 and Nrd1 RRMs recognize different RNA sequences, one intriguing explanation is that Nab3 recognizes important ncRNAs with NNS terminators composed predominantly or exclusively of Nab3 binding sites that Nrd1 cannot recognize in TRAMP mutant cells. On this note, AU-rich sequences (e.g. UAAA; AAAU) and extended Nrd1/Nab3 binding sites have recently been identified in artificial NNS-dependent terminators that are critical for Nrd1-Nab3 interaction, present in native ncRNAs, and could serve as supermotifs for NNS recognition [50]. As Air2 binds to adenosine RNA [15], TRAMP could help Nrd1-Nab3 to cooperatively recognize these AU-rich sequences. If critical ncRNAs contained terminators with AU-rich-Nab3 site supermotifs, this could explain why Nab3, but not Nrd1, specifically suppresses TRAMP mutants. This possibility implies that Nab3 and TRAMP process/degrade a common set of ncRNAs that are critical for cell growth, but are not regulated by Nrd1.

In support of the idea of Nab3- and Nrd1-specific RNA targets, two RNA cross-linking and RNA-Seq studies that mapped Nab3 and Nrd1 sites transcriptome-wide found that a greater percentage of reads for Nab3 RNAs map to RNA Pol II transcripts than that for Nrd1 RNAs (Nab3–73% Pol II vs Nrd1–59% Pol II [23]; Nab3–42% Pol II vs Nrd1–36% Pol II [24]). As yeast cells express more Nrd1 (∼20,000 mols/cell) than Nab3 (∼6,000 mols/cell) [51], this result suggests that Nab3 may bind to more RNA Pol II transcripts than Nrd1. Notably, comparison of the top 100 Nab3 and Nrd1 cross-linked sites from one study reveals that Nab3 cross-links more efficiently to snoRNAs than Nrd1 (Nab3–59/100 sites are snoRNAs vs Nrd1–33/100 sites are snoRNAs [52]). Studies have also reported that the processing/degradation of certain ncRNAs is more prominently altered in *nab3* mutants compared to *nrd1* mutants. In particular, the level of an *FLC1-FMP40* intergenic CUT with three Nab3 binding sites is elevated in *nab3–11* RRM mutant cells, but unchanged in *nrd1–102* RRM mutant cells [25]. The processing of 23S/20S pre-rRNA is also impacted in *nab3–11* cells, but unaltered in *nrd1–102* cells [46]. Finally, the level of 5’-extended pre-tRNA^Arg(UCU)^ is greater in Nab3-depleted cells than it is in Nrd1-depleted cells [23].

The importance of the NNS complex in regulating the exosome, TRAMP, and RNA processing in yeast and conservation of the exosome and TRAMP components in humans raises the question of whether the NNS exosome cofactor is also conserved in humans. In strong support, the human Sen1 helicase, Senataxin, catalyzes termination in human cells [35]. The human SCAF8 RRM protein has also been proposed to be a Nrd1 orthologue based on sequence identity [53]. However, to date, no human Nrd1 or Nab3 orthologue has been functionally characterized. Here, we find that expression of the human RALY (hRALY) RRM-containing protein, which shares homology with Nab3 and contains an RRM with 31% identity to the Nab3 RRM, like Nab3, can improve the growth of *air1/2* TRAMP mutant cells, suggesting that hRALY can modulate a function performed by TRAMP in yeast cells. Consistent with a potential role for hRALY in processing of ncRNAs, nuclear hRALY interacts with numerous RNA binding proteins involved in ncRNA processing/stability in human cells [54].

Combined, the data presented here indicate that Nab3 and Nrd1 can work independently to recruit the exosome to ncRNA targets, providing combinatorial flexibility in RNA processing.

## Materials and Methods

### Chemicals and media

All chemicals were obtained from Sigma-Aldrich (St. Louis, MO), United States Biological (Swampscott, MA), or Fisher Scientific (Pittsburgh, PA) unless otherwise noted. All media were prepared by standard procedures [55].

### Plasmids

All plasmids used in this study are listed in S1 Table. All DNA manipulations were performed according to standard methods [56]. The *URA3 2μ NAB3* (pAC2880), *NRD1* (pAC2869), *SEN1* (pAC3235), *NPL3* (pAC1726), *NAB2* (pAC1813), *HRP1* (pAC1745), and *PUB1* (pAC1759) plasmid were constructed by amplification of *NAB3*, *NRD1*, *SEN1*, *NPL3*, *NAB2*, *HRP1*, and *PUB1* genes by polymerase chain reaction (PCR) from *S*. *cerevisiae* genomic DNA with oligonucleotides (Integrated DNA Technologies) and cloning into pRS426 plasmid [57]. The *URA3 2 μ nab3* RRM mutant *nab3–11* (*F371L-P374L*; pAC2915), *nab3-R331A* (pAC3231), *nab3-F333A* (pAC3232), *nab3-S399A* (pAC3233), and *nab3-S400A* (pAC3234) plasmid, *nab3–1–448* plasmid (pAC3280), and *nab3* Nrd1-binding domain (NBD) mutant *nab3-*Δ*NBD* (Δ*204–248*; pAC3236) plasmid were generated by site-directed mutagenesis with *nab3* oligonucleotides encoding F371L-P374L, R331A, F333A, S399A, S400A, R449X residue substitutions or 204–248 (NBD) residue deletion, *NAB3* (pAC2880) plasmid template, and QuikChange Site-Directed Mutagenesis Kit (Stratagene). The *HIS3 2 μ NAB3* (pAC3246), *nab3* RRM mutants, *nab3–11* (*F371L-P374L*; pAC3247), *nab3-R331A* (pAC3248), *nab3-F333A* (pAC3249), *nab3-S399A* (pAC3250), *nab3-S400A* (pAC3251), *nab3* NBD mutant *nab3-*Δ*NBD* (Δ*204–248*; pAC3252), *NRD1* (pAC3255), *SEN1* (pAC3256), *TRF4* (pAC2940), and *TRF5* (pAC2930) plasmid were constructed by subcloning *NAB3*, *nab3* mutant, *NRD1*, *SEN1*, *TRF4*, *TRF5* genes from pRS426 plasmids into pRS423 [57]. The *URA3 2 μ* C-terminally Myc-tagged *NAB3* (pAC3237), *nab3* RRM mutant *nab3–11* (*P371L-F374L*; pAC3240), *nab3-R331A* (pAC3241), *nab3-F333A* (pAC3242), *nab3-S399A* (pAC3243), and *nab3-S400A* (pAC3244), and *nab3-*Δ*NBD* (Δ204–248; pAC3245) plasmid were constructed by PCR amplification of *NAB3* using oligonucleotides containing 2xMyc tag and *NAB3* (pAC2880), *nab3* RRM mutant (pAC2915, pAC3231–3234) or *nab3-*Δ*NBD* (pAC3236) template and cloning into pRS426. The *URA3 2 μ* C-terminally Myc-tagged *NRD1* (pAC3238), *SEN1* (pAC3239), and *RRP6* (pAC3034) plasmids were constructed by PCR amplification of *NRD1*, *SEN1*, *and RRP6* using oligonucleotides containing 2xMyc tag and *NRD1* (pAC2869), *SEN1* (pAC3235) or W303 gDNA templates and cloning into pRS426. The *LEU2 2 μ* C-terminally TAP-tagged *NAB3* (pAC3253) and *nab3-*Δ*1–248* (pAC3254) plasmids were constructed by PCR amplification of *NAB3* 5’-UTR, full-length *NAB3*, and *nab3 249–802* using oligonucleotides and *NAB3* (pAC2880) plasmid template and cloning into YEp351 plasmid containing C-terminal TAP tag. The *LEU2 2 μ* GFP-tagged *NAB3* (pAC3281) and *nab3–1–448* (pAC3282) plasmids were constructed by PCR amplification of *NAB3* 5’-UTR, full-length *NAB3*, and *nab3–1–448* using oligonucleotides and *NAB3* (pAC2880) template and cloning into YEp351 plasmid containing C-terminal GFP tag. The *URA3* 2 *μ NAB3–5’-UTR-hRALY Isoform 1* (pAC3279) plasmid was constructed by PCR amplification of *NAB3* 5’-UTR using oligonucleotides and *NAB3* (pAC2880) template, PCR amplification of *hRALY* Isoform 1 ORF (GenBank accession number Q9UKM9) using oligonucleotides and human cDNA from HeLa cells, and cloning into pRS426 plasmid. The *URA3 2 μ NAB3–5’-UTR-hRALY-R22A* (pAC3306) and *NAB3–5’-UTR-hRALY-F24A* (pAC3307) mutant plasmids were generated by site-directed mutagenesis with *hRALY* oligonucleotides encoding R22A and F24A residue substitutions and *hRALY* (pAC3279) plasmid template. The *URA3 2 μ* C-terminally Myc-tagged *hRALY* (pAC3308), *hRALY-R22A* (pAC3309), and *hRALY-F24A* (pAC3310) plasmids were constructed by PCR amplification of *NAB3–5’-UTR-hRALY* using oligonucleotides containing 2xMyc tag and *hRALY* (pAC3279), *hRALY-R22A* (pAC3306) or hRALY-F24A (pAC3307) template and cloning into pRS426. The *URA3 CEN6 NAB3* (pAC3285), *NRD1* (pAC3314) and *DIS3* (pAC2861) plasmids were constructed by PCR amplification of *NAB3*, *NRD1*, *and DIS3* genes by PCR from *S*. *cerevisiae* genomic DNA with oligonucleotides and cloning into pRS316 plasmid [58]. The *HIS3 CEN6 AIR1-GFP* (pAC2224) plasmid was constructed by PCR amplification of *AIR1* gene from *AIR1* (pAC1613) plasmid template with oligonucleotides and cloning into pRS313 containing C-terminal GFP tag. The *TRP1 CEN6 RRP6* (pAC2301) plasmid was constructed by PCR amplification of *RRP6* gene from *S*. *cerevisiae* genomic DNA with oligonucleotides and cloning into pRS314 plasmid [58]. The *TRP1 CEN6 rrp6-D238A* (pAC2302) plasmid was generated by site-directed mutagenesis with *rrp6* oligonucleotides encoding D238A residue substitution and pRS314-*RRP6* (pAC2301) plasmid template. The *TRP1 CEN6 dis3-D551N* (pAC2675) plasmid was generated by site-directed mutagenesis with *dis3* oligonucleotides encoding D551N residue substitution and pRS314-*DIS3* plasmid template. The *HIS3 CEN6 nrd1-*Δ*151–214* (pAC3223) plasmid was generated by site-directed mutagenesis with *nrd1* oligonucleotides encoding 151–214 residue deletion and pRS313-*NRD1* plasmid template. The *GST-RRP47* (pAC3311) and *GST-NAB3* (pAC3312) bacterial expression plasmids were constructed by PCR amplification of *NAB3* and *RRP47* genes using oligonucleotides and *NAB3* (pAC2880) or W303 gDNA template and cloning into pGEX-TEV. The *His6-RRP6* (pAC3313) bacterial expression plasmid was constructed by PCR amplification of *RRP6* gene using oligonucleotides and *RRP6* (pAC2301) template and cloning into pET30a-TEV. All constructs were sequenced to ensure the absence of any undesired mutations and the presence of each desired mutation.

### Strains

All strains used in this study are listed in S1 Table. The *air1* (ACY1090), *air2*Δ (ACY1091), and *trf4*Δ (ACY2149) strains were obtained from Research Genetics. The *NRD1-TAP* (ACY2293), *RRP6-TAP* (ACY1063), and *DIS3-TAP* (ACY1926) strains were obtained from Thermo Scientific (Open Biosystems). The *air1-C178R air2*Δ (ACY2020) strain was constructed by insertion of the C178R mutation into the *AIR1* ORF in the W303 strain by the ‘delitto perfetto’ method [59] and deletion of the *AIR2* ORF by homologous recombination with *AIR2-NATMX* PCR product. The *trf4*Δ (ACY2154) strain was constructed by deletion of the *TRF4* ORF in the W303 strain by homologous recombination with *TRF4*-*NATMX* PCR product. The *nab3*Δ (ACY2181) strain was constructed by transformation of a *URA3 NAB3* (pAC3285) plasmid into the W303 strain and deletion of the *NAB3* ORF by homologous recombination with *NAB3*-*NATMX* PCR product. The *air1-C178R air2*Δ *rrp6*Δ strain (ACY2294) was constructed by deletion of the *RRP6* ORF in the ACY2020 strain by homologous recombination with *RRP6-UTR KANMX* PCR product. The *air1-C178R air2*Δ *nrd1*Δ strain (ACY2320) was constructed by transformation of a *URA3 NRD1* (pAC3285) plasmid into the ACY2020 strain and deletion of the *NRD1* ORF by homologous recombination with *NRD1-UTR KANMX* PCR product. The *air1-C178R air2*Δ *dis3*Δ strain (ACY2119) was constructed by transformation of a *URA3 DIS3* (pAC2681) plasmid into the ACY2020 strain and deletion of the *DIS3* ORF by homologous recombination with *DIS3-UTR KANMX* PCR product. The *air1*Δ *air2*Δ (ACY2036) strain was constructed by transformation of a *URA3 AIR2* (pAC1614) plasmid into the W303 strain and consecutive deletion of *AIR1* and *AIR2* ORFs by homologous recombination with *AIR1-NATMX* and *AIR2-HPHMX* PCR products. The *air1-C178R-TAP air2*Δ strain (ACY2051) was constructed by insertion of C-terminal *TAP-Sphis5+* PCR product into the *air1-C178R* ORF in the *air1-C178R air2*Δ (ACY2020) strain by homologous recombination.

### High copy suppressor screen

To identify high copy suppressors of the temperature sensitive growth of *air1-C178R air2*Δ cells at 30°C, *air1-C178R air2*Δ cells (ACY2020) were transformed with a *2 μ URA3* yeast genomic DNA plasmid library and plated on Ura^−^ minimal media plates. As controls, the cells were also transformed with *2 μ URA3* vector (pRS426) or *AIR1* (pAC1613). The cells were grown at 25°C for one day and then shifted 30°C for 2–4 days select for suppressors. Approximately 18,000 transformants containing library plasmids were screened at 30°C. Library plasmids from transformants that showed improved growth at 30°C relative to vector alone were isolated and retransformed into *air1-C178R air2*Δ cells to confirm that these plasmids cause suppression of the thermosensitive growth of the cells at 30°C. The confirmed suppressor plasmids were sequenced to identify the genomic DNA inserts.

### High copy suppression growth assay

To assess suppression of the temperature sensitive growth of *air1-C178R air2*Δ cells, *air1-C178R air2*Δ *nrd1*Δ cells containing *nrd1-*Δ*151–214*, *air1-C178R air2*Δ *rrp6*Δ cells, *air1-C178R air2*Δ *rrp6*Δ cells containing *RRP6* or *rrp6-D238A*, *air1-C178R air2*Δ *dis3*Δ cells containing *dis3-D551N* or the slow growth of Δ*trf4* (ACY2154) cells by TRAMP components, gDNA suppressors, *NAB3*, *nab3* mutants, *NRD1*, *SEN1*, and *NAB/hnRNP*, *hRALY*, *hRALY* mutants, *air1-C178R air2*Δ cells (ACY2020), *air1-C178R air2*Δ *nrd1*Δ (ACY2320) transformed with *nrd1-*Δ*151–214* (pAC3223) *CEN HIS3* plasmid, *air1-C178R air2*Δ *rrp6*Δ (ACY2294), *air1-C178R air2*Δ *rrp6*Δ cells (ACY2294) transformed with *RRP6* (pAC2301) or *rrp6-D238A* (pAC2302) *CEN TRP1* plasmid, *air1-C178R air2*Δ *dis3*Δ cells transformed with *dis3-D551N* (pAC2675) or *trf4*Δ cells (ACY2154) were transformed with vector (pRS426), *AIR1* (pAC1613), *AIR2* (pAC1614), *TRF4* (pAC2147), (*SUP3–2* (pAC3227), *SUP11–3* (pAC3229), *NAB3* (pAC2880), *nab3–11* (pAC2915), *nab3-R331A* (pAC3231), *nab3-F333A* (pAC3232), *nab3-S399A* (pAC3233), *nab3-S400A* (pAC3234), *nab3-*Δ*NBD* (pAC3236), nab3–1–448 (pAC3280), *NRD1* (pAC2869), *SEN1* (pAC3235), *NPL3* (pAC1726), *NAB2* (pAC1813), *HRP1* (pAC1745), *PUB1* (pAC1759), *NAB3 5’UTR-hRALY* Isoform 1 (pAC3279), *NAB3 5’UTR-hRALY-R22A* (pAC3306) or *NAB3 5’UTR-hRALY-F24A* (pAC3307) 2μ *URA3* plasmid and selected on Ura^−^, Ura^−^Trp^−^ or Ura^−^His^−^ minimal media. Cells were grown overnight at 25°C to saturation in Ura^−^, Ura^−^Trp^−^ or Ura^−^His^−^ minimal media, cell concentrations were normalized by OD_600_, and cells were serially diluted and spotted onto Ura^−^, Ura^−^Trp^−^ or Ura^−^His^−^ minimal media plates and grown at 25, 30 and 37°C. To assess bypass suppression of the slow growth of *air1*Δ *air2*Δ cells by *NAB3*, two strains of *air1*Δ *air2*Δ cells (ACY1095, ACY2036), containing *AIR2 URA3* maintenance plasmid (pAC1614), were transformed with vector (pRS423), *AIR1-GFP* (pAC2224), *TRF4* (pAC2940), *TRF5* (pAC2930) or *NAB3* (pAC3246) *2 μ HIS3* plasmids and selected on Ura^−^His^−^ minimal media. Cells were grown overnight at 25°C to saturation in Ura^−^His^−^ minimal media, cell concentrations were normalized by OD_600_, and cells were serially diluted and spotted onto control Ura^−^His^−^ minimal media or His^−^ minimal media containing 5-FOA. Growth of *air1*Δ *air2*Δ cells harboring only *AIR1-GFP*, *TRF4*, *TRF5* or *NAB3* was examined at 25, 30 and 37°C.

### Functional growth assay

To test the *in vivo* function of *nab3* mutants, a standard plasmid shuffle assay combined with serial dilution and spotting was employed. *nab3*Δ cells (ACY2181), containing *NAB3 URA3* maintenance plasmid (pAC3285) were transformed with vector (pRS423), *NAB3* (pAC3246), *nab3–11* (pAC3247), *nab3-R331A* (pA3248), *nab3-F333A* (pAC3249), *nab3-S399A* (pAC3250), *nab3-S400A* (pAC3251), or *nab3-*Δ *NBD* (pAC3252) 2 *μ HIS3* plasmids and selected on Ura^−^His^−^ minimal media. Cells were grown overnight at 25°C to saturation in Ura^−^His^−^ minimal media, cell concentrations were normalized by OD_600_, and cells were serially diluted and spotted onto control Ura^−^His^−^ minimal media, where the *NAB3 URA3* maintenance plasmid is maintained, or His-minimal media containing 5-FOA, which selects for cells that have lost the *NAB3 URA3* maintenance plasmid. Growth of *nab3*Δ cells, harboring *NAB3* or *nab3* mutants as the sole copy of *NAB3*, was examined at 25, 30 and 37°C.

### Analysis of protein expression levels

For analysis of Myc-tagged Nab3, nab3 mutant, Nrd1, and Sen1, hRALY, hRALY mutant protein levels, *air1-C178R air2*Δ cells (ACY2020) expressing Myc-tagged Nab3 (pAC3237), nab3–11 (pAC3240), nab3-R331A (pAC3241), nab3-F333A (pAC3242), nab3-S399A (pAC3243), nab3-S400A (pAC3244), nab3-ΔNBD (pAC3245), Nrd1 (pAC3238) Sen1 (pAC3239), hRALY (pAC3308), hRALY-R22A (pAC3309) or hRALY-F24A (pAC3310) protein were grown in minimal media overnight at 25°C, 10 ml cultures with an OD_600_ = 0.4 were prepared and grown for 2 hr at 25°C, and then shifted to 30°C for 4 hr. For analysis of nab3–1–448 mutant protein level, *air1-C178R air2*Δ cells (ACY2020) containing vector or expressing nab3–1–448 (pAC3280) mutant protein were grown in minimal media overnight at 25°C, 10 ml cultures with an OD_600_ = 0.4 were prepared and grown for 2 hr at 25°C, and then shifted to 30°C for 4 hr. For analysis of TAP-tagged air1-C178R protein levels upon expression of Trf4 or Nab3 protein, *air1-C178R-TAP air2*Δ (ACY2051) cells containing vector (pAC426), *TRF4* (pAC2147) or *NAB3* (pAC2880) were grown in minimal media overnight at 25°C, 10 ml cultures with an OD_600_ = 0.4 were prepared and grown at 25°C for 6 hr. Whole cell lysates of cells were then prepared and 30–50 *μ* g of protein was analyzed by immunoblotting with an anti-Myc monoclonal antibody to detect Myc-tagged proteins, anti-Nab3 monoclonal antibody to detect nab3–1–448, and peroxidase anti-peroxidase antibody to detect TAP-tagged air1-C178R.

### Immunoblotting

Protein samples (30–50 *μ* g lysate; total bound) were resolved on Criterion 4–20% gradient gels (Bio-Rad), transferred to nitrocellulose membranes (Bio-Rad) and Myc-tagged proteins were detected with anti-Myc monoclonal antibody 9B11 (1:2000; Cell Signaling), TAP-tagged proteins were detected with peroxidase anti-peroxidase antibody (1:5000; Sigma), Nrd1 was detected with anti-Nrd1 rabbit polyclonal antibody (1:3,000; a gift from David Brow), Nab3 was detected with anti-Nab3 monoclonal antibody (1:2000; a gift from Maurice Swanson), GFP was detected with anti-GFP rabbit polyclonal antibody (1:3000; Sigma), His-tagged Rrp6 was detected with anti-His monoclonal antibody coupled to horseradish peroxidase (1:2000; Invitrogen), and GST fusion proteins were detected with anti-GST monoclonal antibody (1:2000; Santa Cruz Biotechnology).

### Quantitation of immunoblots and Northern blots

The band intensities/areas from all immunoblots and Northern blots were quantitated using ImageJ v1.4 software (National Institute of Health, MD; http://rsb.info.nih.gov/ij/) and relevant percentages of protein or RNA were calculated in Microsoft Excel for Mac 2011 (Microsoft Corporation). To quantitate the fold overexpression of Nab3 and Nrd1 protein in *air1-C178R air2*Δ cells containing *NAB3* or *NRD1* relative to cells containing vector alone, the Nab3/Nrd1 intensity in cells containing *NAB3* or *NRD1* was normalized to Pgk1 intensity and Nab3/Nrd1 intensity in cells containing vector alone. To quantitate the percentage of air1-C178R-TAP protein in *air1-C178R-TAP air2*Δ cells containing *TRF4* or *NAB3* relative to cells containing vector alone, the air1-C178R-TAP intensity in cells containing *TRF4* or *NAB3* was normalized to Pgk1 intensity and air1-C178R-TAP intensity in cells containing vector alone. To quantitate the percentage of bound nab3-Myc mutant protein relative to bound Nab3-Myc wild-type protein in Nrd1-TAP binding assays, the bound Nab3/nab3-Myc intensity was normalized to bound Nrd1-TAP intensity, input Nab3/nab3-Myc intensity (normalized to Pgk1 intensity), and bound Nab3-Myc wild-type protein intensity. To quantitate the percentage of input nab3-Myc mutant protein relative to input Nab3-Myc wild-type protein, input nab3-Myc intensity were normalized to Pgk1 intensity and Nab3-Myc intensity. To quantitate the percentage of GFP protein in *air1-C178R air2*Δ cells containing pREF-GFP reporter and *NAB3*, *nab3* mutants, *NRD1* or *SEN1*, relative to cells containing pREF-GFP reporter and vector alone in terminator readthrough reporter assays, the GFP intensity in cells containing *NAB3*, *nab3* mutants, *NRD1* or *SEN1* was normalized to Pgk1 intensity and GFP intensity in cells containing vector alone. To quantitate the percentage of *IMD2* CUT and readthrough RNA in W303, *air1-C178R air2*Δ, *trf4*Δ, and *air1-C178R air2*Δ rrp6Δ containing vector, *AIR1*, *TRF4*, *NAB3* or *NRD1* in Northern blots, *IMD2* CUT and readthrough RNA intensity was normalized to *ACT1* or *scR1* RNA intensity and *IMD2* CUT/readthrough intensity in cells containing vector alone. Quantitation refers to specific experiments shown in Figures but is representative of multiple experiments.

### Analysis of RNA expression levels

For analysis of native *IMD2* CUT and readthrough RNA levels, wild-type (W303; ACY233), *air1-C178R air2*Δ (ACY2020), *air1-C178R air2*Δ *rrp6*Δ (ACY2294), and *trf4*Δ (ACY2154) cells containing vector (pRS426), *AIR1* (pAC1613), *TRF4* (pAC2147), *NAB3* (pAC2880), *nab3* mutants (pAC2915, pAC3231–3233, pAC3236) or *NRD1* (pAC2869) were grown in minimal media overnight at 25°C, 10 ml cultures with an OD_600_ = 0.4 were prepared and grown for 2 hr at 25°C, and then shifted to 30°C for 4 hr. Total RNA was isolated from cells and analyzed by Northern blotting with an *IMD2* CUT and *ACT1* or *scR1* loading control oligonucleotide probe (S2 Table).

### Total RNA isolation

To prepare *S*. *cerevisiae* total RNA from cell pellets of 10 ml cultures grown to OD_600_ = 0.5–0.7, glass beads (2–3 x 100 *μ* l) were added to each cell pellet in 2 ml screw-cap tube, 1 ml TRIzol (Invitrogen) was added and cell sample was vigorously disrupted in Mini Bead Beater 16 Cell Disrupter (Biospec) for 2 min at 25°C. For each sample, 100 *μ* l of 1-bromo-3-chloropropane (BCP) was added, the sample was vortexed for 15 sec, and incubated at 25°C for 2 min. Each sample was then centrifuged at 16,300 x *g* for 8 min at 4°C and the upper layer was transferred to a fresh microfuge tube. RNA was precipitated with 500 *μ* l isopropanol and the sample was vortexed for 10 sec to mix. Total RNA was pelleted by centrifugation at 16,300 x *g* for 8 min at 4°C. Supernatant was decanted and 1 ml of 75% ethanol was added to wash RNA pellet. Sample was centrifuged at 16,300 x *g* for 5 min at 4°C. Supernatant was decanted, remaining ethanol was removed, and the RNA pellet was air dried for 15 min. Total RNA was resuspended in 50 *μ* l diethylpyrocarbonate (DEPC (Sigma))-treated water and stored at-80°C.

### Northern blotting

To detect native *IMD2* CUT and readthrough RNA in wild-type, *air1-C178R air2*Δ, *air1-C178R air2*Δ *rrp6*Δ, and *trf4*Δ cells containing vector, *AIR1*, *TRF4*, *NAB3*, *nab3* mutants or *NRD1*, total RNA from cells was resolved on an agarose gel, blotted to a nylon membrane and membrane was probed with radiolabeled *IMD2* CUT and *ACT1* or *scR1* loading control oligonucleotides (S2 Table). Total RNA (10 *μ* g) was mixed with equal volume of RNA loading dye (70% formamide; 2X MOPS; 10% glycerol; 0.1 *μ* g/*μ* l ethidium bromide; 0.03% bromophenol blue) and resolved on 1.2% agarose/1.8% formaldehyde/1X MOPS gel at 70V for 2–3 hr. RNA was blotted to Hybond-N^+^ nylon membrane (Amersham, GE Healthcare) overnight by standard procedure and cross-linked to membrane with UV light (120,000 *μ* Joules) using UV Stratalinker 2400 (Stratagene). Ethidium bromide-stained rRNA on membrane was imaged. Membrane was incubated in Rapid-hyb hybridization buffer (Amersham, GE healthcare) at 42°C for 1 hr. DNA oligonucleotide (100 ng) was 5’-end labeled with [γ-P^32^]-ATP (PerkinElmer) using polynucleotide kinase (New England Biolabs) at 37°C for 30 min. [P^32^]-Labeled oligonucleotide probe was purified through Centrispin-20 spin column (Princeton Separations), heated at 100°C for 5 min, and added to hybridization buffer. Oligonucleotide probe was hybridized to membrane in hybridization buffer at 42°C overnight. Following removal of hybridization buffer, membrane was washed once in 5 x SSC; 0.1% SDS at 25°C for 20 min and washed twice in 1 x SSC; 0.1% SDS at 42°C for 15 min each. Membrane was exposed to phosphoscreen overnight and imaged using Typhoon FLA 7000 phosphoimager (GE Healthcare).

### TAP-tagged protein binding assay

To assess binding between TAP-tagged Nrd1, Rrp6 or Dis3 protein and Myc-tagged Nab3 or nab3 mutant protein or TAP-tagged Nab3 or nab3-Δ1–248 protein and Myc-tagged Rrp6 protein, *NRD1-TAP* (ACY2293), *RRP6-TAP* (ACY1063), *DIS3-TAP* (ACY1926) or untagged BY4741 (ACY1105) cells expressing Myc-tagged Nab3 (pAC3237), nab3 RRM mutants (pAC3240–44), nab3 NBD mutant (pAC3245) or Nrd1 (pAC3238), and W303 cells containing *NAB3-TAP* (pAC3253) or *nab3-* Δ*1–248-TAP* (pAC3254) and expressing Myc-tagged Rrp6 (pAC3034) were inoculated into 50 ml Ura^−^ or Ura^−^Leu^−^ minimal media and grown overnight at 25°C to an OD_600_ = 2. For *NRD1-TAP*, *DIS3-TAP*, and W303 cells, each cell pellet was resuspended in 1 ml IPP150 buffer (10 mM Tris-HCl, pH 8; 150 mM NaCl; 0.1% NP-40) supplemented with protease inhibitors (1 mM phenylmethanesulfonylfluoride (PMSF); 3 ng/ml pepstatin A, leupeptin, aprotinin, and chymostatin). For *RRP6-TAP* and untagged BY4741 cells, each cell pellet was resuspended in 1ml IPP100 buffer (10 mM Tris-HCl, pH 8; 100 mM NaCl; 0.1% NP-40) supplemented with protease inhibitors. To each cell resuspension, glass beads (8 x 100 *μ* l scoops) were added and sample was vigorously disrupted in a Mini Bead Beater 16 Cell Disrupter (Biospec) for 4 x 1 min at 25°C. Protein lysate was cleared by centrifugation at 16,000 x g for 20 min at 4°C. Protein lysate supernatant was transferred to a new microfuge tube, centrifuged at 16,000 x g for an additional 10 min at 4°C, and transferred to a new microfuge tube using a 1 ml syringe. Protein lysate concentration was determined by Bradford assay using Bio-Rad Protein Assay. For Nrd1-TAP, Dis3-TAP, and W303 containing Nab3-TAP samples, protein lysate (2–5 mg) in 1 ml IPP150 buffer was incubated with 25 *μ* l IgG Sepharose beads (GE Healthcare) overnight at 4°C with mixing and beads were washed three times with 1 ml IPP150 buffer for 5 min each. For Rrp6-TAP and untagged BY4741 samples, protein lysate (2–5 mg) in 1 ml IPP100 buffer was incubated with 25 *μ* l IgG Sepharose beads (GE Healthcare) in the presence or absence of 5 *μ* l Purelink RNase A (20 mg/ml; Invitrogen) overnight at 4°C with mixing and beads were washed three times with 1 ml IPP100 buffer for 1 min each. Input lysate (30–50 *μ* g), unbound supernatant (50 *μ* g) and total bound Nrd1-TAP-, Dis3-TAP-, Nab3-TAP-, Rrp6-TAP-, and untagged BY4741-bead sample were analyzed by SDS-PAGE and immunoblotting with anti-Myc monoclonal antibody to detect Nab3-Myc proteins and loading control anti-Pgk1 monoclonal antibody to detect 3-phosphoglycerate kinase.

### Recombinant protein expression and purification

Recombinant GST fusion and His-tagged proteins were expressed in bacteria and purified. GST (pGEX-TEV), GST-Rrp47 (pAC3311), GST-Nab3 (pAC3312), and His-Rrp6 (pAC3313) were expressed in *E*. *coli* DE3 cells and purified by batch purification. Overnight cultures were used to inoculate 100 ml LB media supplemented with 100 *μ* g/ml ampicillin or 50 *μ* g/ml kanamycin. Cultures were grown at 37°C to an OD_600_ of 0.6–0.8, induced with 200 *μ* M IPTG and grown at 25°C overnight. For batch purification of GST fusion proteins, cells were collected and lysed in 10 ml phosphate buffered saline (PBS) supplemented with protease inhibitor mixture (1 mM phenylmethylsulfonyl fluoride, 3 ng/ml pepstatin A, leupeptin, aprotinin, and chymostatin) and 2 mM dithiothreitol (DTT) by incubation with 10 mg lysozyme for 30 min on ice and sonication. Lysates were cleared by centrifugation at 12,000 x *g* for 10 min and incubated with glutathione Sepharose 4B (GE Healthcare) for 2 hr at 4°C with mixing. The beads were then washed once with 10 ml PBS supplemented with 0.5% Triton-X-100 and 2 mM DTT and twice with 10 ml PBS supplemented with 2 mM DTT. GST proteins were eluted from beads with 1 ml 50 mM Tris-HCl, pH 8 containing 10 mM reduced glutathione and dialyzed into PBS supplemented with 2 mM DTT. For batch purification of His-tagged Rrp6 protein, cells were collected and lysed in 10 ml lysis buffer (50 mM NaH_2_PO_4_, pH 7.4, 300 mM NaCl, 10 mM imidazole) supplemented with protease inhibitor mixture and 2 mM DTT by incubation with 10 mg lysozyme and sonication. Lysates were cleared by centrifugation at 12,000 x *g* for 10 min and incubated with nickel-NTA agarose (Qiagen) in lysis buffer for 2 hr at 4°C with mixing. The beads were then washed twice with 10 ml wash buffer (50 mM NaH_2_PO_4_, pH 7.4, 300 mM NaCl, 20 mM imidazole). His-tagged Rrp6 protein was eluted from agarose with 1 ml elution buffer (50 mM NaH_2_PO_4_, pH 7.4, 300 mM NaCl, 250 mM imidazole) and dialyzed into PBS supplemented with 2 mM DTT.

### Recombinant protein binding assay

For GST-Nab3 binding to His-Rrp6, purified soluble GST, GST-Rrp47 or GST-Nab3 (8 *μ* g) was incubated with 14 *μ* g of purified soluble His-Rrp6 and 10 *μ* l glutathione Sepharose 4B (GE Healthcare) in 1 ml IPP100 buffer (10 mM Tris-HCl, pH 8, 100 mM NaCl, 0.1% NP-40) supplemented with 10 mg/ml BSA, 2 mM DTT, and protease inhibitor mixture (0.2 mM phenylmethylsulfonyl fluoride, 3 ng/ml pepstatin A, leupeptin, aprotinin, and chymostatin) for 2 hr at 4C with mixing. Beads were washed three times with 1 ml IPP100 buffer supplemented with 2 mM DTT for 10 sec each. Total bound and 25 *μ* l (2.5%) input samples were analyzed by SDS-PAGE followed by immunoblotting with an anti-His antibody coupled to horseradish peroxidase (Invitrogen) to detect His-Rrp6 and anti-GST antibody (Santa Cruz Biotechnology) to detect GST, GST-Rrp47, and GST-Nab3.

### Terminator readthrough reporter assay

To examine GFP protein expression from the pREF-GFP *IMD2* terminator reporter plasmid in TRAMP mutant cells alone or *air1-C178R air2*Δ cells expressing *NAB3*, *nrd1* mutants, *NRD1*, or *SEN1*, cells containing the pREF-GFP plasmid were grown in the presence of galactose to induce GFP expression, and the level of GFP protein in lysates was detected by immunoblotting. BY4741 Wild-type (ACY402), *air1*Δ (ACY1090), *air2*Δ (ACY1091), *trf4*Δ (ACY2149), W303 wild-type (ACY233), *air1-C178R air2*Δ (ACY2020) cells alone or *air1-C178R air2*Δ (ACY2020) cells containing vector (pAC423), *NAB3* (pAC3246), *nab3–11* (pAC3247), *nab3-R331A* (pAC3248), *nab3-F333A* (pAC3249), *nab3-S399A* (pAC3250), *nab3-S400A* (pAC3251), or *nab3-*Δ*NBD* (pAC3252), *NRD1* (pAC3255) or *SEN1* (pAC3256) *2 μ HIS3* plasmid, were transformed with pREF-GFP (pAC3225; [38]) and selected on Ura^−^ or Ura^−^His^−^ minimal media plates. Cells were grown overnight at 25°C to saturation in Ura^−^ or Ura^−^His^−^ minimal media containing 2% glucose. Cells were then diluted to OD_600_ = 0.4 in 10 ml Ura^−^ or Ura^−^His^−^ minimal media containing 2% raffinose and grown at 30°C for 2 hr. To induce GFP expression from pREF-GFP, 2% galactose was added to cells, 5 ml samples were removed for 0 hr time point, and remaining 5 ml samples were grown at 30°C for 3 hr. Protein lysates from cells at 0 hr and 3 hr time points were analyzed by immunoblotting with an anti-GFP antibody.

### Chromatin immunoprecipitation

To analyze RNA Pol II association with *IMD2* or *snR13* gene in *air1/2* cells, chromatin was prepared from yeast cells and immunoprecipitated with an RNA Pol II antibody according to the protocols of Keogh and Buratowski [60] and Chen *et al*. [61]. The relative Pol II occupancy across *IMD2* or *snR13*-*TRS31* locus was determined by quantitative PCR on chromatin immunoprecipitation and input samples using five *IMD2* or *snR13* primer pairs (S2 Table) and calculation of percentage input. Triplicate 100 ml cultures of *air1-C178R air2*Δ (ACY2020) cells containing vector (pRS426), *AIR1* (pAC1613) or *NAB3* (pAC2880) in Ura^−^ media were grown to OD_600_ = 0.5 at 25°C. *air1-C178R air2*Δ cultures were shifted to 30°C for 1 hr 30 min and grown to OD_600_ = 0.8. Cells were cross-linked with 2.7 ml 37% formaldehyde (1% final) at 25°C for 20 min, incubated with 10 ml glycine stop solution (3 M glycine, 20 mM Tris base) for 5 min at 25°C, collected by centrifugation at 1500 x *g* for 4 min, washed twice with 50 ml TBS (20 mM Tris-HCl, pH 7.5, 150 mM NaCl), washed once with 10 ml FA lysis buffer (50 mM HEPES: KOH, pH 7.5, 150 mM NaCl, 1 mM EDTA, 1% Triton X-100, 0.1% sodium deoxycholate) supplemented with 0.1% SDS, and collected by centrifugation. Cross-linked cell pellets were lysed in 300 *μ* l 300 mM FA Lysis buffer (FA buffer containing 300 mM NaCl) by bead beating. Chromatin extract was sonicated using a Bioruptor sonicator (set to high) for 7 min in 30 sec pulses. Sonicated chromatin extract was centrifuged at 4,000 x *g* for 15 min at 4°C and 500 μg chromatin extract was utilized per immunoprecipitation with an anti-Pol II monoclonal antibody (Clone 4H8; Active Motif). Immune complexes were captured using Protein A-conjugated agarose beads (Santa Cruz Biotechnology) and washed for 5 min each in 300 mM FA lysis buffer, 500 mM FA buffer (FA buffer containing 500 mM NaCl), LiCl buffer (10 mM Tris-HCl, pH 8.0, 250 mM LiCl, 0.5% NP-40, 0.5% sodium deoxycholate, 1 mM EDTA), and TE. Samples were digested with RNase A and eluted from the Protein A-agarose beads in elution buffer (0.1 M sodium bicarbonate, 1% SDS) by two 200 *μ* l room temperature incubations with rotation. IP samples and input samples (10% total chromatin extract) were incubated at 65°C for at least 4 hr to reverse cross-links. DNA was purified using miniprep columns (Qiagen), IP DNA was eluted in 50 *μ* l distilled water, and input DNA in 100 *μ* l distilled water. Quantitative PCR (qPCR) on technical duplicates of IP or input DNA with *IMD2* or *snR13* primers (S2 Table) was performed in 20 *μ* l reactions containing QuantiTect SYBR Green PCR master mix (Qiagen), 0.5 mM primers and 2 *μ* l of IP or input DNA. Each qPCR experiment was performed on a StepOnePlus Real-Time PCR machine (Applied Biosystems) using T_anneal_ = 55°C and 44 cycles. Relative Pol II occupancy was calculated as a percentage of input using the equation: ΔCt = 2^∧^-(IP_Ct_—Input_Ct_). The mean relative occupancy values from three independent experiments were calculated and the mean occupancy values at Primer Pair 1–5 locations were normalized to the value obtained with Primer Pair 1 at the *IMD2* CUT or *snR13* gene in each strain. The normalized mean Pol II occupancy (relative Pol II occupancy) is displayed with error bars that represent the standard error of mean.

### Localization of GFP-tagged proteins

To localize Nab3-GFP and nab3–1–448 proteins, *air1-C178R air2*Δ cells (ACY2020) expressing GFP-tagged Nab3 protein (pAC3281) or nab3–1–448 (pAC3282) were grown in Leu^−^ media with 2% glucose overnight at 25°C, transferred to Ura^−^Leu^−^ media with 2% glucose and grown to log phase at 25°C. GFP fusion proteins were visualized by direct fluorescence microscopy using an Olympus BX60 direct fluorescence microscope equipped with a photometric Quantix digital camera from Roper Scientific (Tucson, AZ) and filters from Chroma Technology (Brattleboro, VT). All images were captured using IP Lab Spectrum software.

## Supporting Information

S1 Fig *NAB3* suppresses the slow growth of *trf4* Δ mutant cells and RNA binding protein genes do not suppress the thermosensitive growth of *air1/2* cells. Related to Figs. 1–2. (**A**) *NAB3* and *nab3-*Δ *NBD* mutant suppress *trf4*Δ slow growth at 25°C, relative to cells containing vector alone, but *nab3–11*, *nab3-R331A*, *nab3-F333A*, and *nab3-S399A* RRM mutants, *NRD1*, and *SEN1* do not suppress *trf4*Δ slow growth at 25°C. The *trf4*Δ cells containing vector, *NAB3*, *nab3* RRM mutants, *nab3-*Δ*NBD* mutant, *NRD1* or *SEN1* 2*μ URA3* plasmid were grown to saturation, serially diluted and spotted on plates, and grown at indicated temperatures. (**B**) RNA binding protein genes, *NPL3*, *HRP1*, *PUB1*, and *NAB2*, do not suppress the thermosensitive growth of *air1-C178R air2*Δ cells at 30°C. The *air1-C178R air2*Δ cells containing vector, *AIR1*, *TRF4*, *GCD14*, *NAB3*, *NPL3*, *HRP1*, *PUB1* or *NAB2* 2 *μ URA3* plasmid were grown to saturation, serially diluted and spotted on plates, and grown at indicated temperatures. See Materials and Methods for details.
(TIF)

S2 Fig Exogenous Nrd1 and Nab3 are overexpressed relative to endogenous Nrd1 and Nab3 in *air1/2* cells. Related to Fig. 1. Lysates of *air1-C178R air2*Δ cells containing vector, *AIR1*, *TRF4*, *NAB3* or *NRD1 2 μ URA3* plasmid at 30°C were analyzed by immunoblotting to detect Nab3 and Nrd1 and 3-phosphoglycerate kinase (Pgk1) as a loading control. Fold overexpression of Nab3 and Nrd1 relative to Pgk1 loading control and cells containing vector alone (Fold Nab3/Nrd1 Rel Vector) is shown below lanes and was calculated as described in Materials and Methods. Nonadjacent lanes in the same immunoblot are separated by white space. Different immunoblots are separated by black boxes.
(TIF)

S3 Fig The nab3 RRM mutant proteins show binding to Nrd1 similar to wild-type Nab3, but the nab3-ΔNBD mutant protein shows greatly reduced binding to Nrd1 and *nab3* RRM and *nab3-*Δ*NBD* mutants do not impair the growth of wild-type cells. Related to Figs. 2–3. **(A)** The nab3-R331A and nab3-S399A RRM mutant proteins show binding to Nrd1 similar to wild-type Nab3, but the nab3-ΔNBD mutant protein shows greatly reduced binding to Nrd1. TAP-tagged Nrd1 was precipitated from lysates of *NRD1-TAP* cells expressing Myc-tagged Nab3, nab3-R331A, nab3-S399A, or nab3-ΔNBD and bound (B), unbound (U), and input fractions were analyzed by immunoblotting to detect Nab3-Myc proteins, Nrd1-TAP proteins and 3-phosphoglycerate kinase (Pgk1) as a loading control. The percentage of bound Nab3 relative to input protein and bound wild-type Nab3 (% Bound) is shown below the bound lanes. The percentage of input Nab3 protein relative to input wild-type Nab3 protein (% Input) is shown below the input lanes. The percentages of protein were calculated as described in Materials and Methods. Quantitation refers to specific experiment shown but is representative of multiple experiments. Immunoblots in Figs. 2B and 3D derive from this original immunoblot. **(B)** Wild-type cells containing vector, *NAB3, nab3–11, nab3-R331A, nab3-F333A, nab3-S399A, nab3-S400A* RRM mutants or *nab3-*Δ*NBD* mutant 2 *μ URA3* plasmid were grown to saturation, serially diluted and spotted on plates, and grown at indicated temperatures. See Materials and Methods for details. Cells spotted in upper and lower panels are on different plates.
(TIF)

S4 Fig *nab3-*Δ*NBD* mutant decreases levels of native *IMD2* CUT and readthrough product similar to *NAB3* in *air1/2* cells, but most *nab3* RRM mutants decrease the level of the *IMD2* CUT and readthrough product to a lesser extent than *NAB3*, and *NAB3* does not significantly affect the termination of native *snR13* snoRNA gene in *air1/2* cells. Related to Fig. 4. **(A)** Northern blot of total RNA from *air1-C178R air2*Δ cells expressing vector, *NAB3*, *nab3-*Δ*NBD* mutant or nab3 RRM mutants, *nab3–11*, *nab3-R331A*, *nab3-F333A*, and *nab3-S399A*, grown at 30°C was probed with an *IMD2* CUT-specific probe. Ethidium bromide-stained 25S rRNA on the Northern blot is shown as a loading control. The *IMD2* CUT (*IMD2* Short/CUT) and *IMD2* CUT readthrough product (*IMD2* Long/RT) are labeled. A longer exposure of *IMD2* CUT readthrough product is shown above. **(B)** *NAB3* does not significantly affect Pol II occupancy downstream of *snR13* gene at Primer Pair 2–5 positions in *air1-C178R air2*Δ cells relative to *air1/2* cells containing vector alone (*p*-value ≥ 0.3), suggesting that Nab3 overexpression does not significantly affect *snR13* termination in *air1/2* cells. Anti-Pol II ChIP was performed on *air1-C178R air2*Δ cells containing vector or *NAB3* and relative Pol II occupancy was measured within and downstream of *snR13* gene by qPCR with *snR13* Primer Pair 1–5 as described in Material and Methods. Mean RNA Pol II occupancy values from three independent experiments normalized to Primer Pair 1 within *snR13* gene are shown with error bars that represent standard error of the mean. Statistical significance of differences in mean Pol II occupancy values was determined using unpaired *t* test. Schematic of *snR13* gene and downstream *TRS31* gene is shown with positions of *snR13* qPCR Primer Pairs 1–5 above and base pair distances between primer pairs below.
(TIF)

S5 Fig *NAB3* does not suppress *air1/2 rrp6*Δ cells expressing the catalytically inactive rrp6 mutant, rrp6-D238A, and Rrp6 binds to nab3-Δ1–248 NBD mutant in a similar manner to wild-type Nab3. Related to Fig. 5. (**A**) *NAB3* does not suppress the thermosensitive growth of *air1/2 rrp6*Δ cells expressing the catalytically inactive rrp6 Mutant, rrp6-D238A, at 30°C. *air1-C178R air2*Δ *rrp6*Δ cells containing *RRP6* or *rrp6-D238A* and vector, *AIR1*, *AIR2*, *TRF4*, *NAB3*, *NRD1* or *SEN1* 2 *μ URA3* plasmid were grown to saturation, serially diluted and spotted on plates, and grown at indicated temperatures. See Supplemental Experimental Procedures for details. Cells spotted in upper and lower panels are on different plates. (**B**) Rrp6 binds to nab3-Δ1–248 NBD mutant in a similar manner to wild-type Nab3. TAP-tagged Nab3 or nab3-Δ1–248 mutant protein was from lysates of wild-type cells expressing NAB3-TAP or nab3-Δ1–248-TAP and Myc-tagged Rrp6 and bound (B), unbound (U), and input fractions were analyzed by immunoblotting with an anti-Myc antibody to detect Rrp6-Myc proteins and an anti-Pgk1 antibody to detect 3-phosphoglycerate kinase (Pgk1) as a loading control. See Materials and Methods for details. Nonadjacent lanes in the same immunoblot are separated by white space. Different immunoblots are separated by black boxes.
(TIF)

S6 Fig Alignment of Nab3 and hRALY full-length protein sequences. hRALY RNA recognition motif (RRM) has 31% identity with the Nab3 RRM and hRALY C-terminal domain has 11% identity with the Nab3 C-terminal domain. Single RRM domains are boxed and RRM consensus motifs RNP1 and RNP2 are marked with lines above. Identical residues are shaded in black and similar residues are shaded in gray. Residue numbers are shown on left. Nab3 and hRALY Isoform 1 (GenBank accession number Q9UKM9) protein sequences were aligned with ClustalW2 sequence alignment tool and shaded using BoxShade software.
(TIF)

S7 Fig The *nab3–1–448* mutant containing the Nab3 RRM but lacking the C-terminal domain does not suppress *air1/2* thermosensitive growth, even though this mutant is expressed and localized to the nucleus. Related to Fig. 6 (**A**) *nab3–1–448* mutant that retains RRM but lacks C-terminal domain of Nab3 does not suppress the thermosensitive growth of *air1/2* cells. *air1-C178R air2*Δ cells containing vector, *NAB3*, *nab3–11*, or *nab3–1–448* 2μ *URA3* plasmid were grown to saturation, serially diluted and spotted on plates, and grown at indicated temperatures. (**B**) nab3–1–448 mutant protein is expressed in *air1/2* cells. Lysates of *air1-C178R air2*Δ cells containing vector or expressing nab3–1–448 mutant at 30°C were analyzed by immunoblotting with anti-Nab3 antibody to detect Nab3 protein and anti-Nrd1 antibody to detect Nrd1 as a loading control. Endogenous Nab3 and overexpressed nab3–1–448 are detected by anti-Nab3 antibody. (**C**) nab3–1–448 mutant protein localizes to the nucleus like Nab3 in *air1/2* cells. *air1-C178R air2* cells expressing C-terminally GFP-tagged Nab3 or nab3–1–448 mutant were visualized by direct fluorescence microscopy. DAPI stain shows the position of the nucleus. Differential interference contrast (DIC) images visualize the cells. See Materials and Methods for details.
(TIF)

S1 Table Yeast strains and plasmids used in this study. (DOCX)

S2 Table DNA oligonucleotides used in this study. (DOCX)
