# Supplementary figures and images for: Nab3 Facilitates the Function of the TRAMP Complex in RNA Processing via Recruitment of Rrp6 Independent of Nrd1
Source: PLoS Genet. 2015 Mar 16;11(3):e1005044. doi: 10.1371/journal.pgen.1005044 (PMC4361618; doi:10.1371/journal.pgen.1005044)

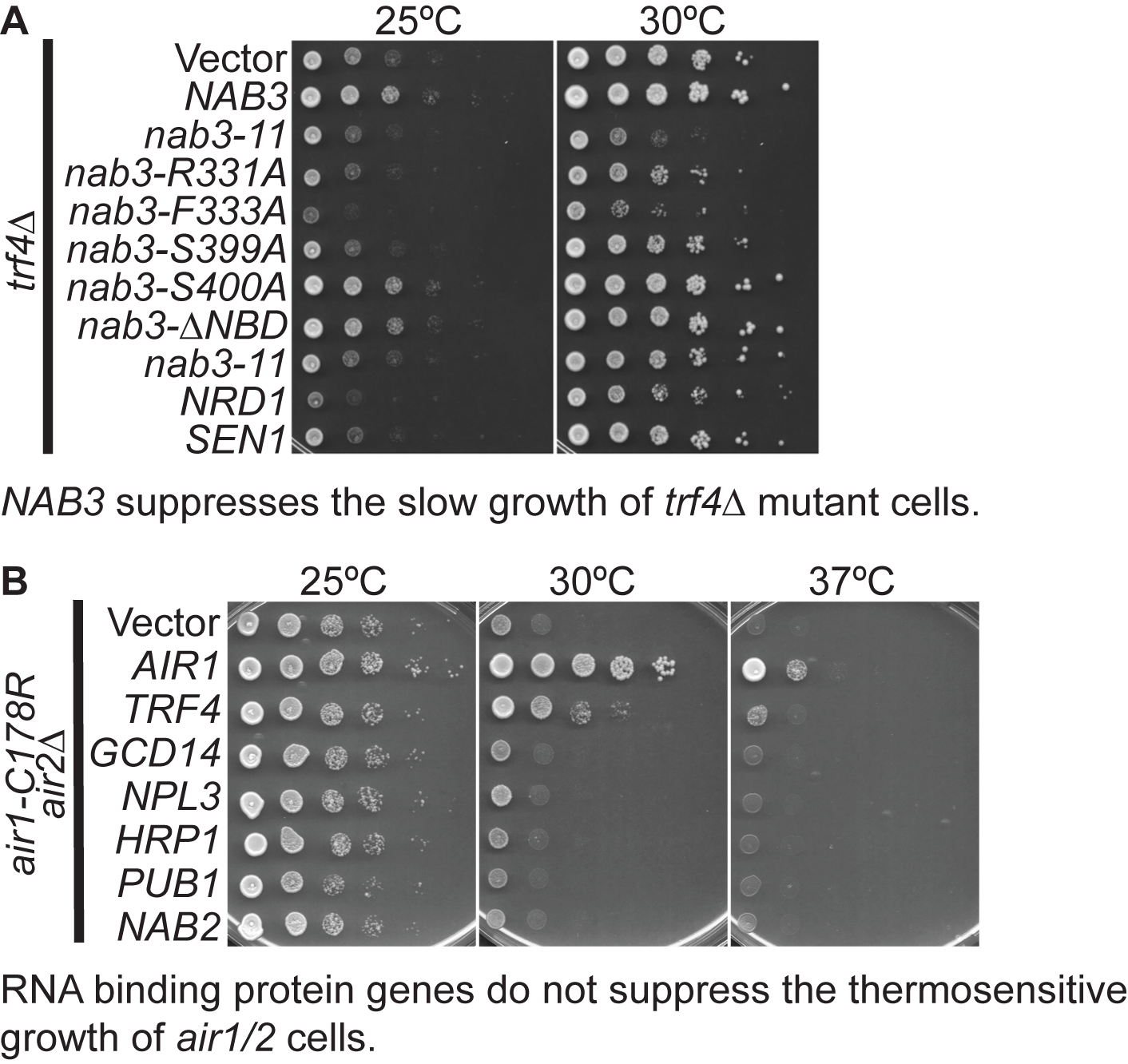

Supplement: S1 Fig — Related to Figs. 1–2. (A) NAB3 and nab3-Δ NBD mutant suppress trf4Δ slow growth at 25°C, relative to cells containing vector alone, but nab3–11, nab3-R331A, nab3-F333A, and nab3-S399A RRM mutants, NRD1, and SEN1 do not suppress trf4Δ slow growth at 25°C. The trf4Δ cells containing vector, NAB3, nab3 RRM mutants, nab3-ΔNBD mutant, NRD1 or SEN1 2μ URA3 plasmid were grown to saturation, serially diluted and spotted on plates, and grown at indicated temperatures. (B) RNA binding protein genes, NPL3, HRP1, PUB1, and NAB2, do not suppress the thermosensitive growth of air1-C178R air2Δ cells at 30°C. The air1-C178R air2Δ cells containing vector, AIR1, TRF4, GCD14, NAB3, NPL3, HRP1, PUB1 or NAB2 2 μ URA3 plasmid were grown to saturation, serially diluted and spotted on plates, and grown at indicated temperatures. See Materials and Methods for details. (TIF) [file pgen.1005044.s001.tif]

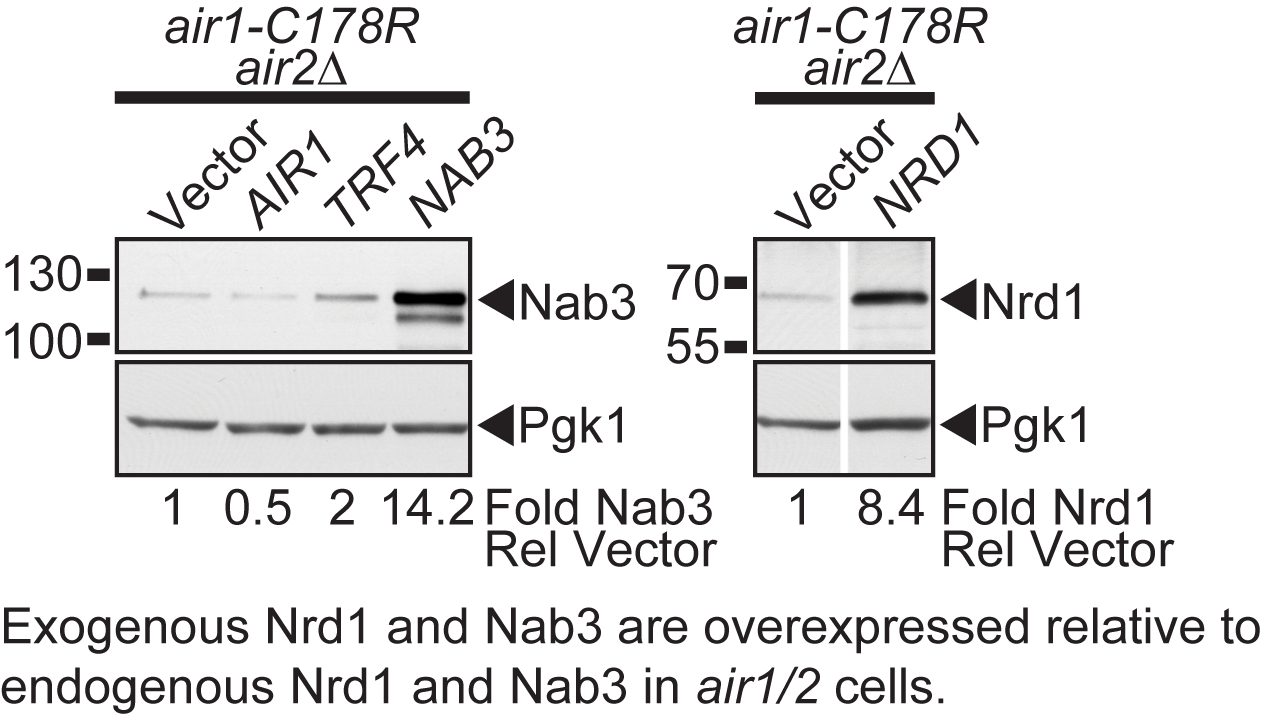

Supplement: S2 Fig — Related to Fig. 1. Lysates of air1-C178R air2Δ cells containing vector, AIR1, TRF4, NAB3 or NRD1 2 μ URA3 plasmid at 30°C were analyzed by immunoblotting to detect Nab3 and Nrd1 and 3-phosphoglycerate kinase (Pgk1) as a loading control. Fold overexpression of Nab3 and Nrd1 relative to Pgk1 loading control and cells containing vector alone (Fold Nab3/Nrd1 Rel Vector) is shown below lanes and was calculated as described in Materials and Methods. Nonadjacent lanes in the same immunoblot are separated by white space. Different immunoblots are separated by black boxes. (TIF) [file pgen.1005044.s002.tif]

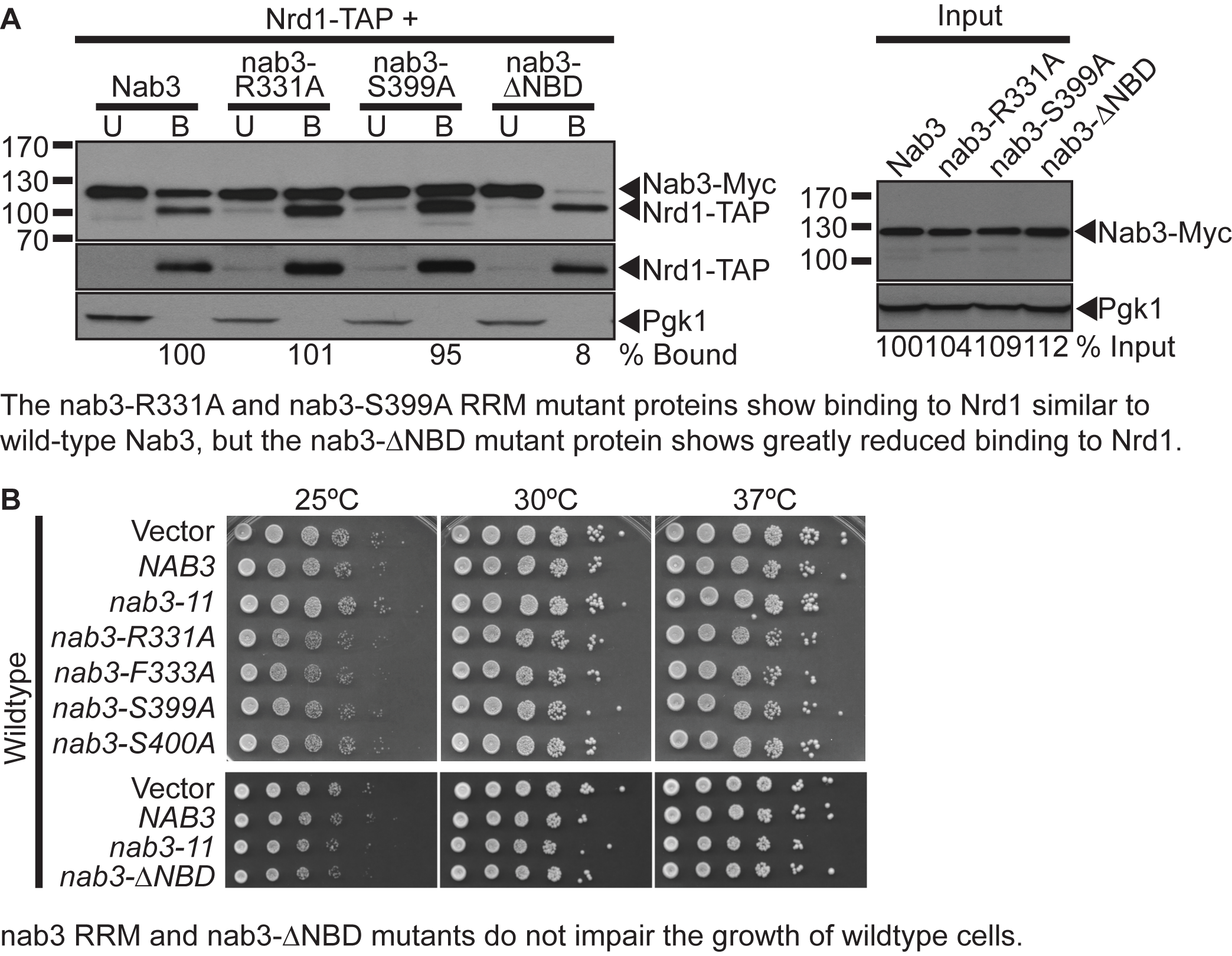

Supplement: S3 Fig — Related to Figs. 2–3. (A) The nab3-R331A and nab3-S399A RRM mutant proteins show binding to Nrd1 similar to wild-type Nab3, but the nab3-ΔNBD mutant protein shows greatly reduced binding to Nrd1. TAP-tagged Nrd1 was precipitated from lysates of NRD1-TAP cells expressing Myc-tagged Nab3, nab3-R331A, nab3-S399A, or nab3-ΔNBD and bound (B), unbound (U), and input fractions were analyzed by immunoblotting to detect Nab3-Myc proteins, Nrd1-TAP proteins and 3-phosphoglycerate kinase (Pgk1) as a loading control. The percentage of bound Nab3 relative to input protein and bound wild-type Nab3 (% Bound) is shown below the bound lanes. The percentage of input Nab3 protein relative to input wild-type Nab3 protein (% Input) is shown below the input lanes. The percentages of protein were calculated as described in Materials and Methods. Quantitation refers to specific experiment shown but is representative of multiple experiments. Immunoblots in Figs. 2B and 3D derive from this original immunoblot. (B) Wild-type cells containing vector, NAB3, nab3–11, nab3-R331A, nab3-F333A, nab3-S399A, nab3-S400A RRM mutants or nab3-ΔNBD mutant 2 μ URA3 plasmid were grown to saturation, serially diluted and spotted on plates, and grown at indicated temperatures. See Materials and Methods for details. Cells spotted in upper and lower panels are on different plates. (TIF) [file pgen.1005044.s003.tif]

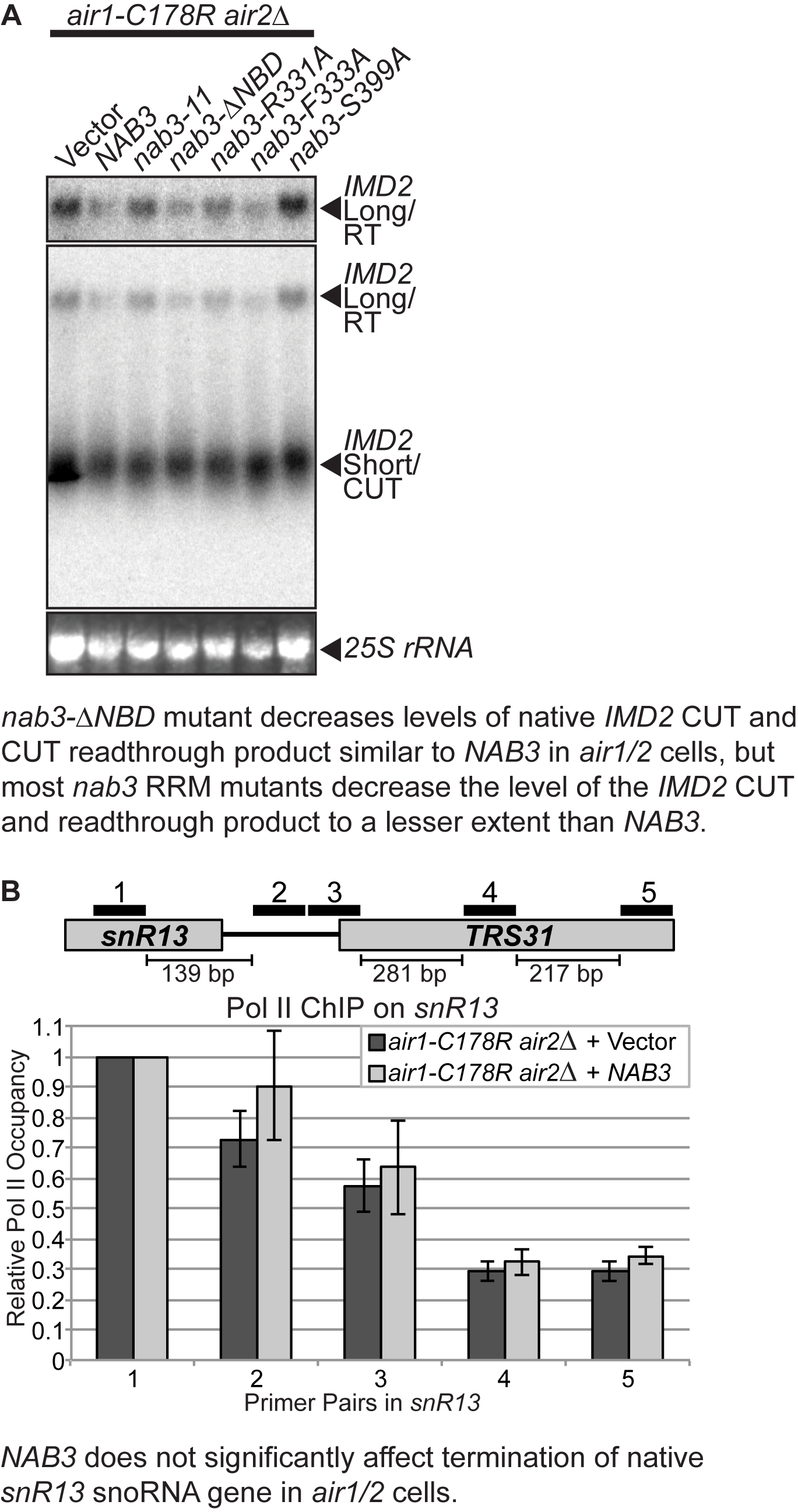

Supplement: S4 Fig — Related to Fig. 4. (A) Northern blot of total RNA from air1-C178R air2Δ cells expressing vector, NAB3, nab3-ΔNBD mutant or nab3 RRM mutants, nab3–11, nab3-R331A, nab3-F333A, and nab3-S399A, grown at 30°C was probed with an IMD2 CUT-specific probe. Ethidium bromide-stained 25S rRNA on the Northern blot is shown as a loading control. The IMD2 CUT (IMD2 Short/CUT) and IMD2 CUT readthrough product (IMD2 Long/RT) are labeled. A longer exposure of IMD2 CUT readthrough product is shown above. (B) NAB3 does not significantly affect Pol II occupancy downstream of snR13 gene at Primer Pair 2–5 positions in air1-C178R air2Δ cells relative to air1/2 cells containing vector alone (p-value ≥ 0.3), suggesting that Nab3 overexpression does not significantly affect snR13 termination in air1/2 cells. Anti-Pol II ChIP was performed on air1-C178R air2Δ cells containing vector or NAB3 and relative Pol II occupancy was measured within and downstream of snR13 gene by qPCR with snR13 Primer Pair 1–5 as described in Material and Methods. Mean RNA Pol II occupancy values from three independent experiments normalized to Primer Pair 1 within snR13 gene are shown with error bars that represent standard error of the mean. Statistical significance of differences in mean Pol II occupancy values was determined using unpaired t test. Schematic of snR13 gene and downstream TRS31 gene is shown with positions of snR13 qPCR Primer Pairs 1–5 above and base pair distances between primer pairs below. (TIF) [file pgen.1005044.s004.tif]

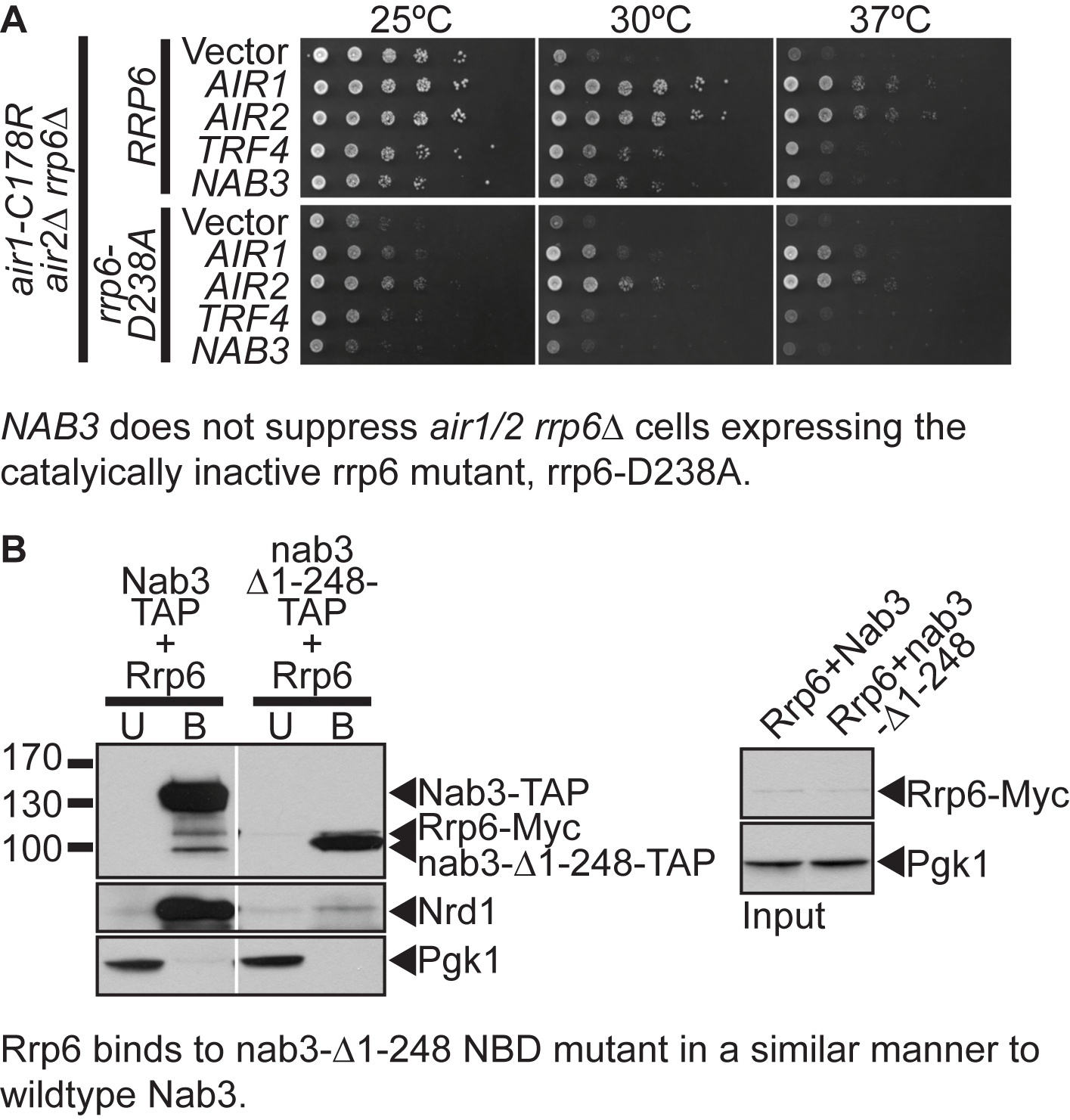

Supplement: S5 Fig — Related to Fig. 5. (A) NAB3 does not suppress the thermosensitive growth of air1/2 rrp6Δ cells expressing the catalytically inactive rrp6 Mutant, rrp6-D238A, at 30°C. air1-C178R air2Δ rrp6Δ cells containing RRP6 or rrp6-D238A and vector, AIR1, AIR2, TRF4, NAB3, NRD1 or SEN1 2 μ URA3 plasmid were grown to saturation, serially diluted and spotted on plates, and grown at indicated temperatures. See Supplemental Experimental Procedures for details. Cells spotted in upper and lower panels are on different plates. (B) Rrp6 binds to nab3-Δ1–248 NBD mutant in a similar manner to wild-type Nab3. TAP-tagged Nab3 or nab3-Δ1–248 mutant protein was from lysates of wild-type cells expressing NAB3-TAP or nab3-Δ1–248-TAP and Myc-tagged Rrp6 and bound (B), unbound (U), and input fractions were analyzed by immunoblotting with an anti-Myc antibody to detect Rrp6-Myc proteins and an anti-Pgk1 antibody to detect 3-phosphoglycerate kinase (Pgk1) as a loading control. See Materials and Methods for details. Nonadjacent lanes in the same immunoblot are separated by white space. Different immunoblots are separated by black boxes. (TIF) [file pgen.1005044.s005.tif]

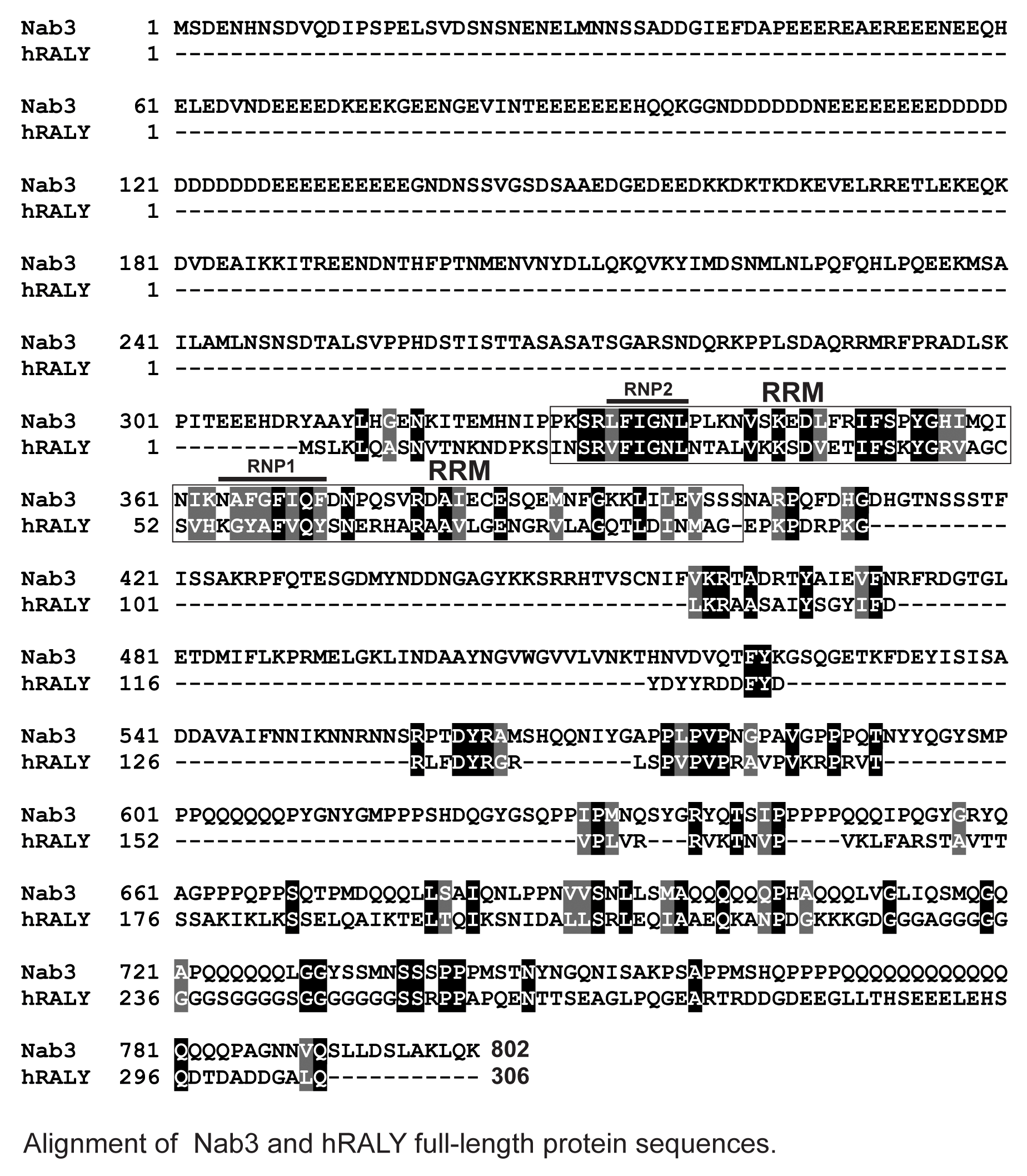

Supplement: S6 Fig — hRALY RNA recognition motif (RRM) has 31% identity with the Nab3 RRM and hRALY C-terminal domain has 11% identity with the Nab3 C-terminal domain. Single RRM domains are boxed and RRM consensus motifs RNP1 and RNP2 are marked with lines above. Identical residues are shaded in black and similar residues are shaded in gray. Residue numbers are shown on left. Nab3 and hRALY Isoform 1 (GenBank accession number Q9UKM9) protein sequences were aligned with ClustalW2 sequence alignment tool and shaded using BoxShade software. (TIF) [file pgen.1005044.s006.tif]

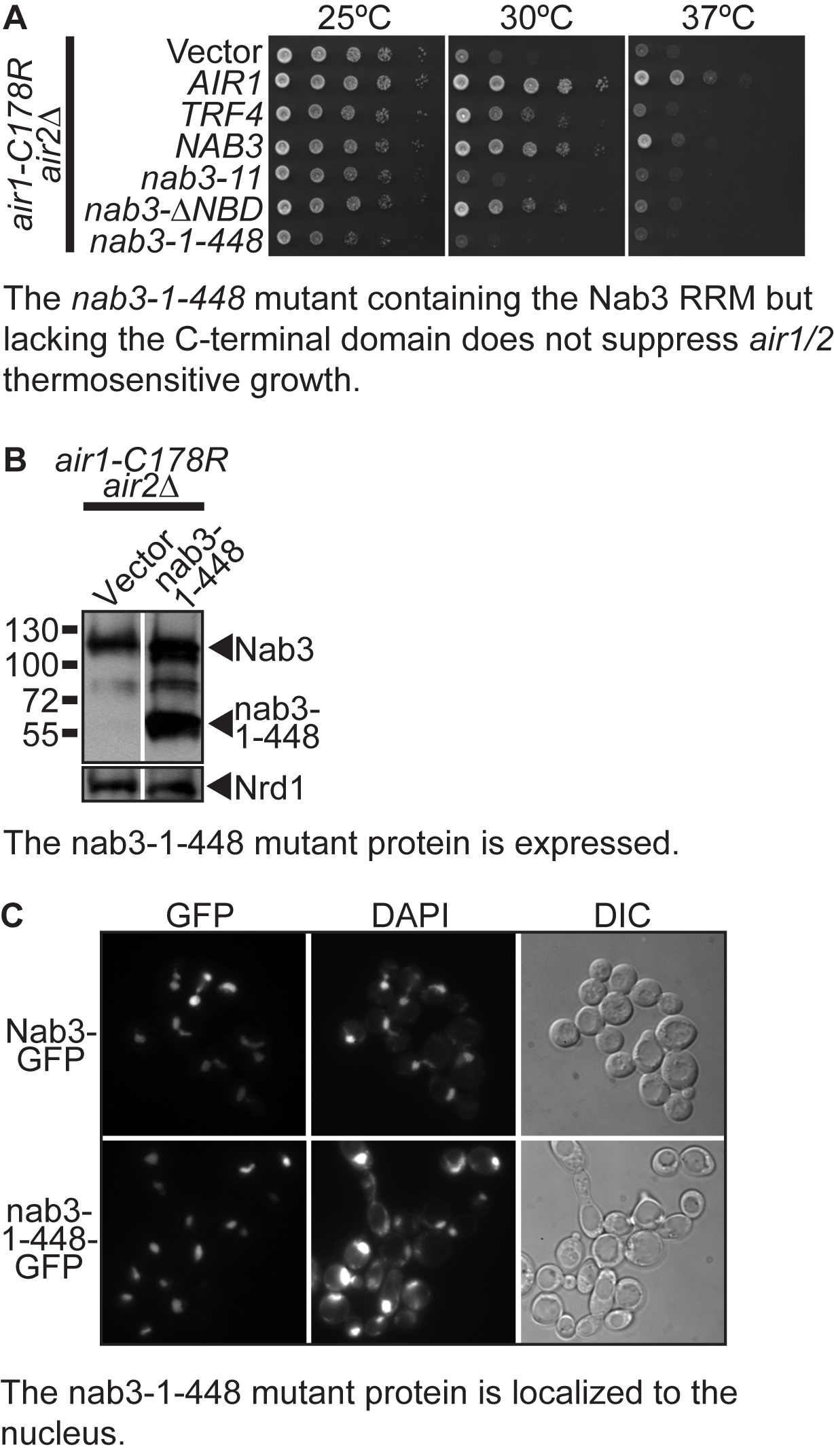

Supplement: S7 Fig — Related to Fig. 6 (A) nab3–1–448 mutant that retains RRM but lacks C-terminal domain of Nab3 does not suppress the thermosensitive growth of air1/2 cells. air1-C178R air2Δ cells containing vector, NAB3, nab3–11, or nab3–1–448 2μ URA3 plasmid were grown to saturation, serially diluted and spotted on plates, and grown at indicated temperatures. (B) nab3–1–448 mutant protein is expressed in air1/2 cells. Lysates of air1-C178R air2Δ cells containing vector or expressing nab3–1–448 mutant at 30°C were analyzed by immunoblotting with anti-Nab3 antibody to detect Nab3 protein and anti-Nrd1 antibody to detect Nrd1 as a loading control. Endogenous Nab3 and overexpressed nab3–1–448 are detected by anti-Nab3 antibody. (C) nab3–1–448 mutant protein localizes to the nucleus like Nab3 in air1/2 cells. air1-C178R air2 cells expressing C-terminally GFP-tagged Nab3 or nab3–1–448 mutant were visualized by direct fluorescence microscopy. DAPI stain shows the position of the nucleus. Differential interference contrast (DIC) images visualize the cells. See Materials and Methods for details. (TIF) [file pgen.1005044.s007.tif]
