# Supplementary material for: Nab3 Facilitates the Function of the TRAMP Complex in RNA Processing via Recruitment of Rrp6 Independent of Nrd1
Source: PLoS Genet. 2015 Mar 16;11(3):e1005044. doi: 10.1371/journal.pgen.1005044 (PMC4361618; doi:10.1371/journal.pgen.1005044)
Supplement: S1 Table — (DOCX) [file pgen.1005044.s008.docx]

S1 Table -Yeast Strains and Plasmids

| Strain/Plasmid | Description | Source |
| --- | --- | --- |
| W303 wild-type (ACY233) | *MATα ura3Δ leu2Δ trp1Δ his3Δ* |  |
| BY4741 wild-type (ACY402) | *MATa leu2∆ ura3∆ his3∆ TRP1 met15∆* | RG* |
| BY4741 wild-type (ACY1105) | *MATa leu2∆ ura3∆ his3∆ TRP1 met15∆* | TS* |
| *air1Δ air2Δ* (ACY1095) | *MATa leu2Δ ura3Δ his3Δ TRP1 AIR1::KAN AIR2::KAN* (pAC1614) *BY4741* | [1] |
| *air1Δ air2Δ* (ACY2036) | *MATa leu2Δ ura3Δ his3Δ TRP1 AIR1::NAT AIR2::HPH* (pAC1614) *W303* | This Study |
| *air1∆* (ACY1090) | *MATa leu2∆ ura3∆ his3∆ TRP1 AIR1::KAN BY4741* | RG* |
| *air2∆* (ACY1091) | *MATa leu2∆ ura3∆ his3∆ TRP1 AIR2::KAN BY4741* | RG* |
| *trf4∆* (ACY2149) | *MATa leu2∆ ura3∆ his3∆ TRP1 TRF4::KAN BY4741* | RG* |
| *trf4∆* (ACY2154) | *MATα ura3Δ leu2Δ trp1Δ his3Δ TRF4::NAT W303* | This Study |
| *nab3∆* (ACY2181) | *MATα ura3Δ leu2Δ trp1Δ his3Δ NAB3::NAT W303* (pAC3285) | This Study |
| *air1-C178R air2Δ* (ACY2020) | *MATα ura3Δ leu2Δ trp1Δ his3Δ air1-C178R AIR2::NAT W303* | [1] |
| *air1-C178R air2∆ nrd1∆* (ACY2320) | *MATα ura3Δ leu2Δ trp1Δ his3Δ air1-C178R AIR2::NAT NRD1::KAN W303*(pAC3285) | This Study |
| *air1-C178R air2Δ rrp6∆* (ACY2294) | *MATα ura3Δ leu2Δ trp1Δ his3Δ air1-C178R AIR2::NAT RRP6::KAN W303* | This Study |
| *air1-C178R air2Δ dis3∆* (ACY2119) | *MATα ura3Δ leu2 Δtrp1Δhis3Δ air1-C178R AIR2::NAT DIS3::KAN W303*(pAC2681) | This Study |
| *air1-C178R-TAP air2Δ* (ACY2051) | *MATα ura3Δ leu2Δ trp1Δ his3Δair1-C178R-TAP:Sphis5+AIR2::NAT W303* | This Study |
| *NRD1-TAP* (ACY2293) | *MATa ura3Δ leu2Δ his3Δ NRD1-TAP:Sphis5+ BY4741* | TS* |
| *RRP6-TAP* (ACY1063) | *MATa ura3Δ leu2Δ his3Δ RRP6-TAP:Sphis5+ BY4741* | TS* |
| *DIS3-TAP* (ACY1926) | *MATa ura3Δ leu2Δ his3Δ DIS3-TAP:Sphis5+ BY4741* | TS* |
|  |  |  |
| pRS423 | *2µ, HIS3, AMP^R^* | [2] |
| pRS426 | *2µ, URA3, AMP^R^* | [2] |
| YEp351 | *2µ, LEU2, AMP^R^* | [3] |
| pRS313 | *CEN6, HIS3, AMP^R^* | [4] |
| pRS314 | *CEN6, TRP1, AMP^R^* | [4] |
| pRS316 | *CEN6, URA3, AMP^R^* | [4] |
| pGEX-TEV | *GST-TEV, AMP^R^, Bacterial Expression Vector* | [5] |
| pET30a-TEV | *His_6_-TEV, KAN^R^, Bacterial Expression Vector* | [6] |
| pREF-GFP (pAC3225) | *IMD2-IT-GFP, GAL, 2µ, URA3, AMP^R^* | [7] |
| pAC1613 | *AIR1, CEN6, URA3, AMP^R^* | [1] |
| pAC1614 | *AIR2, CEN6, URA3, AMP^R^* | [1] |
| pAC2147 | *TRF4, 2µ, URA3, AMP^R^* | [1] |
| pAC3227 | *SUP3-2 (AIR1), 2µ, URA3, AMP^R^* | This Study |
| pAC3229 | *SUP11-3 (NAB3), 2µ, URA3, AMP^R^* | This Study |
| pAC2880 | *NAB3, 2µ, URA3, AMP^R^* | This Study |
| pAC2915 | *nab3-11(F371L, P374L), 2µ, URA3, AMP^R^* | This Study |
| pAC3231 | *nab3-R331A, 2µ, URA3, AMP^R^* | This Study |
| pAC3232 | *nab3-F333A, 2µ, URA3, AMP^R^* | This Study |
| pAC3233 | *nab3-S399A, 2µ, URA3, AMP^R^* | This Study |
| pAC3234 | *nab3-S400A, 2µ, URA3, AMP^R^* | This Study |
| pAC3236 | *nab3-∆NBD(∆204-248), 2µ, URA3, AMP^R^* | This Study |
| pAC3280 | *nab3-1-448, 2µ, URA3, AMP^R^* | This Study |
| pAC1726 | *NPL3, 2µ, URA3, AMP^R^* | This Study |
| pAC1813 | *NAB2, 2µ, URA3, AMP^R^* | This Study |
| pAC1745 | *HRP1, 2µ, URA3, AMP^R^* | This Study |
| pAC1759 | *PUB1, 2µ, URA3, AMP^R^* | This Study |
| pAC2869 | *NRD1, 2µ, URA3, AMP^R^* | This Study |
| pAC3235 | *SEN1, 2µ, URA3, AMP^R^* | This Study |
| pAC3279 | *NAB3-5’-UTR-hRALY Isoform 1, 2µ, URA3, AMP^R^* | This Study |
| pAC3306 | *NAB3-5’-UTR-hRALY-R22A, 2µ, URA3, AMP^R^* | This Study |
| pAC3307 | *NAB3-5’-UTR-hRALY-F24A, 2µ, URA3, AMP^R^* | This Study |
|  |  |  |
|  |  |  |
|  |  |  |
| Strain/Plasmid | Description | Source |
| pAC3237 | *NAB3-2xMyc, 2µ, URA3, AMP^R^* | This Study |
| pAC3240 | *nab3-11(F371L, P374L)-2xMyc, 2µ, URA3, AMP^R^* | This Study |
| pAC3241 | *nab3-R331A-2xMyc, 2µ, URA3, AMP^R^* | This Study |
| pAC3242 | *nab3-F333A-2xMyc, 2µ, URA3, AMP^R^* | This Study |
| pAC3243 | *nab3-S399A-2xMyc, 2µ, URA3, AMP^R^* | This Study |
| pAC3244 | *nab3-S400A-2xMyc, 2µ, URA3, AMP^R^* | This Study |
| pAC3245 | *nab3-∆NBD(∆204-248)-2xMyc, 2µ, URA3, AMP^R^* | This Study |
| pAC3238 | *NRD1-2xMyc, 2µ, URA3, AMP^R^* | This Study |
| pAC3239 | *SEN1-2xMyc, 2µ, URA3, AMP^R^* | This Study |
| pAC3034 | *RRP6-2xMyc, 2µ, URA3, AMP^R^* | This Study |
| pAC3308 | *NAB3-5’-UTR-hRALY-2xMyc*, *2µ, URA3, AMP^R^* | This Study |
| pAC3309 | *NAB3-5’-UTR-hRALY-R22A-2xMyc*, *2µ, URA3, AMP^R^* | This Study |
| pAC3310 | *NAB3-5’-UTR-hRALY-F24A-2xMyc*, *2µ, URA3, AMP^R^* | This Study |
| pAC3246 | *NAB3, 2µ, HIS3, AMP^R^* | This Study |
| pAC3247 | *nab3-11(F371L, P374L), 2µ, HIS3, AMP^R^* | This Study |
| pAC3248 | *nab3-R331A, 2µ, HIS3, AMP^R^* | This Study |
| pAC3249 | *nab3-F333A, 2µ, HIS3, AMP^R^* | This Study |
| pAC3250 | *nab3-S399A, 2µ, HIS3, AMP^R^* | This Study |
| pAC3251 | *nab3-S400A, 2µ, HIS3, AMP^R^* | This Study |
| pAC3252 | *nab3-∆NBD(∆204-248), 2µ, HIS3, AMP^R^* | This Study |
| pAC3255 | *NRD1, 2µ, HIS3, AMP^R^* | This Study |
| pAC3256 | *SEN1, 2µ, HIS3, AMP^R^* | This Study |
| pAC2940 | *TRF4, 2µ, HIS3, AMP^R^* | This Study |
| pAC2930 | *TRF5, 2µ, HIS3, AMP^R^* | This Study |
| pAC2224 | *AIR1-GFP, CEN6, HIS3, AMP^R^* | This Study |
| pAC3285 | *NAB3, CEN6, URA3, AMP^R^* | This Study |
| pAC2301 | *RRP6, CEN6, TRP1, AMP^R^* | This Study |
| pAC2302 | *rrp6-D238A, CEN6, TRP1, AMP^R^* | This Study |
| pAC2681 | *DIS3, CEN6, URA3, AMP^R^* | This Study |
| pAC2675 | *dis3-D551N, CEN6, TRP1, AMP^R^* | This Study |
| pAC3314 | *NRD1, CEN6, URA3, AMP^R^* | This Study |
| pAC3223 | *nrd1-∆151-214, CEN6, HIS3, AMP^R^* | This Study |
| pAC3253 | *NAB3-TAP, 2µ, LEU2, AMP^R^* | This Study |
| pAC3254 | *nab3-∆1-248-TAP, 2µ, LEU2, AMP^R^* | This Study |
| pAC3281 | *NAB3-GFP, 2µ, LEU2, AMP^R^* | This Study |
| pAC3282 | *nab3-1-448-GFP, 2µ, LEU2, AMP^R^* | This Study |
| pAC3311 | *GST-RRP47 in pGEX-TEV, AMP^R^* | This Study |
| pAC3312 | *GST-NAB3 in GEX-TEV, AMP^R^* | This Study |
| pAC3313 | *His6-RRP6 in pET30a-TEV, KAN^R^* | This Study |

TS*-Thermo Scientific

RS* - Research Genetics

Table S1 References

1. Fasken MB, Leung SW, Banerjee A, Kodani MO, Chavez R, et al. (2011) Air1 Zinc Knuckles 4 and 5 and a Conserved IWRXY Motif Are Critical for the Function and Integrity of the Trf4/5-Air1/2-Mtr4 Polyadenylation (TRAMP) RNA Quality Control Complex. J Biol Chem 286: 37429-37445.

2. Christianson TW, Sikorski RS, Dante M, Shero JH, Hieter P (1992) Multifunctional yeast high-copy-number shuttle vectors. Gene 110: 119-122.

3. Hill JE, Myers AM, Koerner TJ, Tzagoloff A (1986) Yeast/E. coli shuttle vectors with multiple unique restriction sites. Yeast 2: 163-167.

4. Sikorski RS, Hieter P (1989) A system of shuttle vectors and yeast host strains designed for efficient manipulation of DNA in *Saccharomyces cerevisiae*. Genetics 122: 19-27.

5. Matsuura Y, Stewart M (2004) Structural basis for the assembly of a nuclear export complex. Nature 432: 872-877.

6. Fairall L, Chapman L, Moss H, de Lange T, Rhodes D (2001) Structure of the TRFH dimerization domain of the human telomeric proteins TRF1 and TRF2. Mol Cell 8: 351-361.

7. Loya TJ, O'Rourke TW, Reines D (2012) A genetic screen for terminator function in yeast identifies a role for a new functional domain in termination factor Nab3. Nucleic Acids Res 40: 7476-7491.
