# Supplementary material for: Nab3 Facilitates the Function of the TRAMP Complex in RNA Processing via Recruitment of Rrp6 Independent of Nrd1
Source: PLoS Genet. 2015 Mar 16;11(3):e1005044. doi: 10.1371/journal.pgen.1005044 (PMC4361618; doi:10.1371/journal.pgen.1005044)
Supplement: S2 Table — (DOCX) [file pgen.1005044.s009.docx]

S2 Table - DNA Oligonucleotides

| Description | | Sequence (5’-3’) | Name | |  |
| --- | --- | --- | --- | --- | --- |
| Northern probes: | |  |  | |  |
| *IMD2* CUT Rev primer | | GTATTATTGAAATTGGTTATATGGAAAATAATAAGAAAAGTAAGGC | AC4745 | |  |
| *ACT1* Rev primer | | ACGTGAGTAACACCATCACCGGAA | AC3284 | |  |
| *scR1* Rev primer | | ATCCCGGCCGCCTCCATCAC | AC5936 | |  |
|  | |  |  | |  |
| ChIP qPCR primers: | |  |  | |  |
|  | |  |  | |  |
| *IMD2* Fwd primer 1 | | TTTTGCTGAACATTTAACCGGAG | AC6635 | |  |
| *IMD2* Rev primer 1 | | AAAAGGTTCCCAGCATACTAGG | AC6636 | |  |
| *IMD2* Fwd primer 2 | | TCCACAAGTAGCAAAAGCAATG | AC6637 | |  |
| *IMD2* Rev primer 2 | | TCTTGGTAGGCTCTTGGTAAAG | AC6638 | |  |
| *IMD2* Fwd primer3 | | GGAAAAGTATGGATTTGCAGGC | AC6639 | |  |
| *IMD2* Rev primer 3 | | TCCTGAACGAGTAAAGAGTTGTC | AC6640 | |  |
| *IMD2* Fwd primer 4 | | TGCTCAAGTGGGTCAAAGAG | AC6641 | |  |
| *IMD2* Rev primer 4 | | AGCCAGTTCCCATACCAATTC | AC6642 | |  |
| *IMD2* Fwd primer 5 | | CTACCTCCCGTTACTTTTCCG | AC6643 | |  |
| *IMD2* Rev primer 5 | | TGGATCCTTTGTCAACGACAG | AC6644 | |  |
| *snR13* Fwd Primer 1 | TGGCATCTCAAATCGTCTCTATATC | | | AC6602 | |
| *snR13* Rev Primer 1 | CACACCGTTACTGATTTGGC | | | AC6603 | |
| *snR13* Fwd Primer 2 | CGTTGGGTTTGGCTTGTTC | | | AC6604 | |
| *snR13* Rev Primer 2 | GTACTCCCTGTCTTTCTGTCG | | | AC6605 | |
| *snR13* Fwd Primer 3 | ACAGGGAGTACTATCACCATCC | | | AC6606 | |
| *snR13* Rev Primer 3 | GCTTCGCTTGTTATCGCTTG | | | AC6607 | |
| *snR13* Fwd Primer 4 | CAAATGCGTCAAATTCCCCG | | | AC6608 | |
| *snR13* Rev Primer 4 | CTGTTTGCTTTTCTTGGAGTCG | | | AC6609 | |
| *snR13* Fwd Primer 5 | CGTTTGCGGCATTATCAAAGG | | | AC6610 | |
| *snR13* Rev Primer 5 | ATCTCAAACCTTCCCTGTCAAG | | | AC6611 | |
